# Cannabis use patterns, prevalence, and risk factors in Nigeria: a systematic review and meta-analysis

**DOI:** 10.1186/s42238-025-00337-0

**Published:** 2025-11-11

**Authors:** Ogochukwu W. Odeigah, Emeka W. Dumbili, Ediomo-Ubong Nelson

**Affiliations:** 1 Department of Psychology, Chrisland University, K/M 5 Ajebo Road, Abeokuta, Ogun State Nigeria; 2 School of Sociology, University College Dublin, Dublin, Ireland; 3 Centre for Research and Information On Substance Abuse, Uyo, Nigeria

**Keywords:** Cannabis, Systematic review, Meta-analysis, Nigeria

## Abstract

**Background:**

Reviews on cannabis use have been conducted in high-income countries, but limited data exist in Sub-Saharan Africa. In Nigeria, cannabis use is widespread, but systematic reviews summarizing the available data to generate robust evidence that can facilitate interventions have not been conducted. This systematic review and meta-analysis synthesized results from studies highlighting the patterns, prevalence, risk factors and motivations for cannabis use in Nigeria.

**Methods:**

Databases searched include PubMed, Web of Science, Scopus, CINAHL, PsycINFO, ProQuest, African Journal Online (AJOL), and Google Scholar, for articles published in English between January 2000 and December 2023. Search terms were developed and slightly modified for each database. The primary outcomes were studies that examined the patterns, prevalence, risk factors, or motivation for cannabis use in Nigeria. The PECO framework guided the review, and all of the studies included were assessed for methodological rigor using the Joanna Briggs Institute checklist for prevalence studies.

**Results:**

The search yielded 1,911 research articles, which were screened, resulting in 72 studies being included in the review, with a total sample size of 45,968. Among adolescents, lifetime and past one-month prevalence of cannabis use were 3.90% (95% confidence interval (CI) = 1.99–7.51; *I*^2^ = 97.6), and 2.34% [95% CI = 1.17–4.61; *I*^2^ 96.1). Among adults, lifetime and past one-month prevalence of cannabis use were 8.28% (95% CI = 4.60–14.46; *I*^2^ = 99.3) and 6.51% (95% CI = 3.48–11.87; *I*^2^ = 97.2). Lifetime and one-month prevalence of cannabis use were 9.93% (95% CI = 4.36–21.08; *I*^2^ = 99.6) and 3.56% (95% CI = 1.65–7.54; *I*^2^ = 96.2) in the Northern region, and 5.59% (95% CI = 2.78–10.91; *I*^2^ = 96.9) and 2.90% (95% CI = 1.27–6.47; *I*^2^ = 97.2) in Western regions. Being a male was a risk factor for cannabis use and was mostly reported by studies of adults, and secondary school and university students. Cannabis was obtained from different sources, including peers, vendors and open markets.

**Conclusion:**

Cannabis use prevalence is high in Nigeria, and several factors that may increase cannabis related harm are the motivating factors. The findings show the need for prevention programs targeting young people and treatment services for problematic users.

**Supplementary Information:**

The online version contains supplementary material available at 10.1186/s42238-025-00337-0.

## Introduction

Cannabis is considered the most widely used illegal drug across the world, with 228 million people estimated in 2022 to have used the drug in the past year globally (United 2024). The available data on cannabis use prevalence are mostly from high-income countries in North America and Europe, where cannabis use prevalence is monitored through routine population-based surveys (Legleye et al. 2014; Peacock et al. 2018). Africa is experiencing an increase in cannabis use, especially among young people, with more people seeking treatment for cannabis-related problems than any other drug (Economics 2024). Yet, less is known about cannabis use prevalence in Africa, with the exception of a recent systematic review estimating the lifetime prevalence of cannabis use among adolescents and adults in the sub-Saharan region (9% and 12.6%, respectively) (Belete et al. 2023). However, the review (Belete et al. 2023) was based on fifty-three studies, most of which were conducted in Nigeria and South Africa. Relatedly, the review focused only on cannabis use prevalence. Therefore, further studies —particularly country-specific studies—are needed to better understand usage patterns (e.g., lifetime, past 12-months, past one-month, past 7-days, and daily use), and prevalence rates (i.e., proportion of the population affected), including risk factors (such as demographic, behavioral, or contextual variables) associated with an increased likelihood of, or vulnerability to, cannabis use.

A comprehensive synthesis of studies examining cannabis use in Nigeria is necessary for two main reasons. First, the prior review (Belete et al. 2023) included only 12 studies conducted in Western Nigeria between 2010 and 2020. Second, 11 of the studies included in the review focused solely on adolescents aged 10–17 years in school settings. This narrow scope fails to capture the broader landscape of cannabis use across Nigeria’s diverse population and geography. With Nigeria’s 36 states spread across Western, Southern, Eastern, and Northern regions, such a limited geographic and demographic focus is a major gap. A systematic scoping of peer-reviewed and grey literature reveals that during and since the review period, primary studies have examined cannabis use across varied age groups and populations in Nigeria. For example, during the same period as when the review was conducted, an additional 35 peer-reviewed primary studies exploring the prevalence of cannabis use across various populations and age groups were published. Between 2021 and 2024, 20 more studies were published, with 3 examining risk factors associated with cannabis use and 12 focusing on specific populations such as university undergraduates, commercial drivers, and individuals who were internally displaced or incarcerated. The studies also cover previously underrepresented regions, including the Northern (9 studies), Western (7 studies), Eastern (4 studies) and Southern (1 study) regions.

Cannabis is the most widely used drug classified as illegal in Nigerian laws. It is commonly known as “Igbo”, “weed”, “marijuana”, “Indian hemp”, and “ganja”, in Nigeria, “dagga” in South Africa, and “bhang” in Kenya and Uganda. The national survey of drug use in Nigeria estimated that 10.6 million people (10.8% of the population) aged 15–64 years used cannabis, while 1 in 3 were dependent on the drug (United 2019). The survey also estimated the mean age of initiation for cannabis use among the general population at 19 years, with the highest prevalence observed among young people within the age range of 25 to 39 years. Other studies, including those using qualitative methods, corroborate the general impression of an increase in cannabis use among young people and also explore the motivations and other social and structural factors shaping consumption patterns (Dirisu et al. 2019; Dumbili 2020; Dumbili et al. 2021; Nelson 2021a; Nelson 2022; Nelson 2024; Ugwu 2022).

Cannabis use, especially regular use, is associated with a range of negative effects, including impairment of cognitive and behavioral functions and risk of psychiatric disorders (Hall, 2009a). Early cannabis use initiation and regular use of high-potency cannabis, especially during adolescence, have been linked to an elevated risk of negative social, health and behavioral outcomes such as cannabis use disorders, comorbid psychiatric conditions, academic difficulties and persistent developmental challenges in adulthood (Bagot 2015; Hall 2009b). Understanding the prevalence of cannabis use and the factors that shape its patterns of use is important given the potential harms, but the current evidence is based on small-scale studies and is therefore of limited utility in regard to the design of intervention strategies. What is needed are systematic reviews that summarize the existing research to highlight trends in cannabis use prevalence across social groups. The findings could inform the development and tailoring of intervention strategies as well as the identification of relevant topics and hypotheses to be examined through further research.

In this study, we conducted a systematic review and meta-analysis of existing studies that examined the prevalence, patterns, and risk factors for cannabis use in Nigeria. Nigeria is a good case for examining cannabis use prevalence for a range of reasons, notably its large population size and also the availability of fairly recent national prevalence data that has been the basis for the estimation of drug use prevalence on the African continent in recent years (United 2019). Local cannabis cultivation is widespread in Nigeria (Abikoye et al. 2019), and the country has been a central drug trafficking route in West Africa, despite punitive policies criminalizing cannabis use (Klantschnig 2016; Molobe 2021). Hence, imported/trafficked cannabis (and other substances) contributes to fueling the drug availability, use and related harms in Nigeria.

A comprehensive review of cannabis use in Nigeria should date back to the 2000 s and not 2010, as was the case with the previous review. From 1994 to 2000, Nigeria implemented a highly punitive drug policy, largely influenced by international pressure to crack down on drug trafficking and use (Obot 2004). This period saw the peak of “Operation Burn the Weeds,” a national cannabis eradication campaign that focused on eradication and punishment, reaching its height in 1999 with the destruction of approximately 3,500 hectares of cannabis farms Mostly in the southwest (Obot 2004). The country also introduced the National Drug Control Masterplan in 1999 with the vision to actualize a drug free society (National 1999). In 2000, the national prevalence of cannabis abuse among individuals aged 15 to 64 was 13.8%, alongside 272,260 kg recorded seizures of cannabis herb—a figure that increased significantly to 683,101 kg by 2004 (United 2007).

Two decades later, cannabis is still classified as an illegal substance in Nigeria, while its possession, cultivation and trafficking can result in severe penalties, including imprisonment ranging from 15 to 25 years (Federal 2004). Although there has been efforts to implement alternatives to incarceration for possession of small quantities of cannabis, Nigeria continues to maintain a strict prohibitionist stance on cannabis with no current legal framework for decriminalization or regulated use, despite growing momentum towards reform (Nelson 2025; Nelson 2022). Enforcement is often characterized by repressive strategies (e.g., frisking) with regular surveillance and crackdowns by the National Drug Law Enforcement Agency (NDLEA).

The current study aims to answer the research question: ‘‘what are the patterns, and prevalence of cannabis use in Nigeria, and what risk factors are most consistently associated with use?” The specific objective of this systematic review and meta-analysis was to synthesize primary studies that examine the patterns, prevalence and risk factors of cannabis use among the general population in Nigeria. The review addressed the gaps identified in the earlier review and the findings provide information on existing patterns of cannabis use, differences across population groups and the underlying risk factors shaping its prevalence in Nigeria.Specifically, our review includes: 1) the identification of cannabis use patterns across a wider population (adolescents vs. adults); 2) the synthesis of risk factors that may inform future theoretical frameworks on cannabis use in low-resource settings; and 3) the identification of measurement inconsistencies and gaps. These insights also provide evidence that can inform policy development and guide the implementation of targeted harm reduction strategies.

## Materials and method

We conducted a systematic review and meta-analysis using the Preferred Reporting Items for Systematic Reviews and Meta-Analysis (PRISMA) (Moher et al. 2009) guideline. The PRISMA checklist provides guidance on the transparency and quality of reporting systematic reviews and meta-analyses. The checklist contains 27 items that are presented in a manner that reflects the structure of a research paper. The main research question for the review was: “what are the patterns, and prevalence of cannabis use in Nigeria, and what risk factors are most consistently associated with use?” The protocol for the review is registered at the International Register of Systematic Reviews (PROSPERO), with the registration number CRD42023413986 (Odeigah 2024). PROSPERO is a database for prospectively registering systematic reviews and is aimed at improving transparency, reducing reporting bias and preventing unintended research duplication (Schiavo 2019).

### Search strategy and databases

We searched electronic databases that index peer-reviewed journal articles such as PubMed, Web of Science, Scopus, Cumulative Index to Nursing and Allied Health Literature (CINAHL), PsycINFO, ProQuest, African Journal Online (AJOL), and Google Scholar. PubMed, CINAHL, and PsycINFO are specialized databases and index studies related to health and behavioral sciences. Web of Science and Scopus are multidisciplinary citation databases that index a broad range of peer-reviewed literature across the sciences, social sciences, and humanities. African Journals Online (AJOL) hosts peer-reviewed journals from across Africa, while Google Scholar and ProQuest provide broader access to diverse research. Searches were conducted in the title and abstract fields of each database using search terms developed and slightly modified for each database and are provided in Supplementary Table S1. The search was conducted in April 2024 and updated in June 2024 to ensure that we had included recent research, but our inclusion criteria were restricted to studies published between January 2000 and December 2023.

### Selection criteria

To determine eligibility, we used the population, exposure, comparison, and outcome (PECO) checklist (Tacconelli 2010). The PECO checklist and framework is a structured approach used to formulate research questions and guide study selection, inclusion/exclusion criteria and the interpretation of findings for systematic reviews. The population consisted of individuals of all ages in Nigeria, the exposure was cannabis use, and the outcomes were usage patterns (e.g., lifetime, past 12-months, past one-month, past 7-days, and daily use) and prevalence rates (i.e., proportion of the population affected), including risk factors (such as demographic, behavioral, or contextual variables), and motivation for use. Two authors conducted the initial article search and screened the articles based on the titles and abstracts of the studies. Studies included in the review had to be peer-reviewed, have collected original quantitative data from human participants, be written in English, and have been published between January 2000 and December 2023. Studies were required to have examined the prevalence, risk factors, or motivations for cannabis use among males and females across all age groups. Only quantitative studies were included. Studies that examined other new psychoactive substances in addition to cannabis were eligible if only the prevalence, risk factors, motivations or source of cannabis was identifiable. Articles that were outside the inclusion criteria were excluded by consensus between two of the authors. Articles that met the inclusion criteria were retained for further assessment. The full text of the remaining articles was downloaded for quality assessment to determine the studies that would be included in the final analysis.

### Methodological assessment and risk of bias

The quality of each study was assessed using the Joanna Briggs Institute (JBI) Critical Appraisal Checklist for studies reporting prevalence data (Munn 2014). The JBI checklist is used to assess the methodological rigor and risk of bias of studies reporting prevalence data for use in systematic reviews. The checklist has 9 questions which assesses sampling, measurement and analysis. The 9 questions in the checklist include: (Q1) Was the sample frame appropriate to address the target population?’; (Q2) Were study participants recruited in an appropriate way?; (Q3) Was the sample size adequate?; (Q4) Were the study subjects and setting described in detail?; (Q5) Was the data analysis conducted with sufficient coverage of the identified sample?; (Q6) Were valid methods used for the identification of the condition?; (Q7) Was the condition measured in a standard, reliable way for all participants?; (Q8) Was there appropriate statistical analysis?; and (Q9) Was the response rate adequate, and if not, was the low response rate managed appropriately?. The response format for each question and its score is yes = 1, no = 0, unclear or not/applicable = 0. Based on previous work (Belete et al. 2023) the total score on each study was calculated as the total number of ‘yes’ responses obtained. Studies scoring 7–9 were categorized as having low risk of bias; 4—6 as having moderate risk; and 0—3 as having high risk. Two authors assessed the studies included. Differences in appraisal were evaluated until a consensus was reached. The resulting Cohen’s κ was 0.71, indicating substantial agreement. There were no disagreements between ratings requiring resolution by a third reviewer. Supplementary Table S2 contains the methodological quality and risk of bias of the studies included in the review.

### Data extraction

The data extraction from the included studies was done by one author and reviewed by a second author to ensure consistency and accurateness. The data extracted included the study authors, year and aim; participants’ characteristics (study population, sample size and age); study setting (city, State/region); measures, other substances studied, and term adopted for cannabis; and the results (prevalence data, risk factors and motivation for cannabis use). The data was extracted into a Microsoft Excel file.

### Statistical analysis

We conducted a meta-analysis using the DerSimonian-Laird random-effects model in *R software* (version 4.5.1). We estimated the pooled prevalence of cannabis use across five patterns of use: i) lifetime, ii) past 12 months, iii) past one month, iv) past 7 days, and v) daily. These calculations were stratified by population group (adolescents and adults). For each category, pooled prevalence estimates with 95% confidence intervals were reported. To assess the heterogeneity across studies, we calculated Cochran’s Q statistic, the *I*^2^ index, and τ^2^ estimates. Subgroup analyses were conducted when sufficient studies were available within each subgroup. Each study was treated as a separate unit of analysis per reported pattern of cannabis use (e.g., lifetime, past-year, past-month etc.). If a single study reported multiple patterns, each pattern was extracted and analyzed independently, ensuring that the prevalence estimates were specific to the defined use category. This approach allowed us to synthesize the data across studies while maintaining conceptual clarity and avoiding duplication within any single prevalence estimate.

### Publication bias

Publication bias was assessed through visual inspection of funnel plots and statistical testing using Egger’s regression and Begg–Mazumdar’s rank correlation. Should substantial publication bias be detected, the classic fail-safe N test was utilized to estimate the number of unpublished or missing studies necessary for the observed publication bias to exceed the threshold for statistical non-significance (i.e., *p* > 0.05).

## Results

**Fig. 1:**
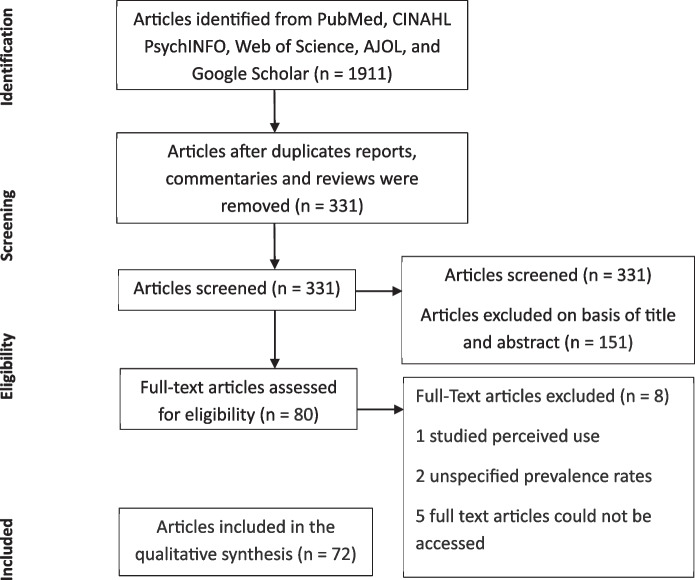
PRISMA flow diagram on studies identified through systematic literature search and included in the review

Figure 1 summarizes the PRISMA flow diagram of articles identified through a systematic literature search and included in the review. The initial search yielded 1911 articles, which were reduced to 331 after removing duplicates, reviews, and reports. The titles and abstracts of the 331 articles were examined, and 151 articles were removed because they did not meet the eligibility criteria. The full texts of the remaining 80 articles with primary data were reviewed and 8 were removed because they were found to not have met the eligibility criteria. The final 72 articles (Supplementary Table S3) investigated the patterns, prevalence, risk factors, and motivations of cannabis, marijuana, Indian hemp use, including sources of cannabis.

Almost all the studies (*n* = 71) examined the prevalence of cannabis, marijuana and Indian hemp alongside other psychoactive substances, and they were mainly cross-sectional surveys. Thirteen studies independently assessed risk factors associated with the use of cannabis, marijuana and Indian hemp, while two studies each assessed motivations for use and sources of supply.

### Study characteristics, designs and measures

Table 1 contains a summary of the characteristics of the studies reviewed. The sample size of the 72 studies ranged from 70 to 6752, while the total sample across all the studies was 45, 968. Most of the studies were conducted in Western (*n* = 28) and Northern (*n* = 25) states in Nigeria. Eight studies were conducted in Eastern states, and 10 in Southern states.

**Table 1:** Summary of studies reviewed on patterns and prevalence of cannabis use in Nigeria

Characteristics	Number of Studies
Population	
Adolescents	31
Adults	41
Region	
Northern	25
Eastern	8
Southern	10
Western	28
Patterns of Cannabis use	
Daily	6
Past 7 day	7
Past one month	40
Past 12 Months	19
Lifetime	42
Risk factors	13
Motivation for use	2

Across the studies, 58.3% reported lifetime use of cannabis (*n* = 42), 26.4% reported past 12 months use (*n* = 19), 55.6 reported past one month use (*n* = 40), 5.6% reported past 7 days use (*n* = 4) and 8.3% reported daily use (*n* = 6). Four studies sampled only male participants, while 2 sampled only female participants. Participants were mostly secondary school (*n* = 25) and university (*n* = 19) students. Other studies were conducted among psychiatric patients (*n* = 6), commercial drivers (*n* = 5), and adolescents (*n* = 4). Three studies each were conducted among people living with HIV, primary health care patients, and incarcerated persons. Two studies were conducted among adults (18–65 + years) and internally displaced persons. One study each sampled street children, pregnant women, military officers, informal religious school children (Almajiris), and people who used drugs.

The studies were generally of high quality (*n* = 58), 18% were moderate and 1 was considered weak. Probability sampling methods were mostly adopted (*n* = 55, 72.4%), and 87% studies (*n* = 63) reported a response rate of above 90%. Only one study did not report a response rate. Studies mostly used the terms cannabis (*n* = 37) and marijuana (*n* = 19). Nine studies used the term Indian hemp, while one study used hashish. Some studies used a combination of terms such as cannabis and marijuana (*n* = 1), hashish and marijuana (*n* = 1), marijuana and Indian hemp (*n* = 1), and cannabis, marijuana, and Indian hemp (*n* = 1). Most of the studies (*n*= 70) examined cannabis use alongside other psychoactive substances. Only one study (Shehu 2008) investigated marijuana use and associated factors among a sample of secondary school students. The instruments used to measure cannabis use varied across the studies. They included a semi-structured questionnaire developed by the authors (*n* = 33), the WHO student substance use survey questionnaire (*n* = 18), the Alcohol, Smoking and Substance Involvement Screening Test (ASSIST, *n* = 6), the WHO Composite International Diagnostic Interview (CIDI, *n* = 3), and the Drug use Disorders Identification Test (DUDIT, *n* = 2). Other instruments used included the youth risk behavior survey (*n* = 2), Drug Abuse Screening Test (DAST, *n* = 1), health-related quality of life SF-12 (*n* = 1), Car, Relax, Alone, Forget, Friends, Trouble (CRAFFT) (*n* = 1), and others (*n* = 2).

### The pattern and prevalence of cannabis use

**Fig. 2:**
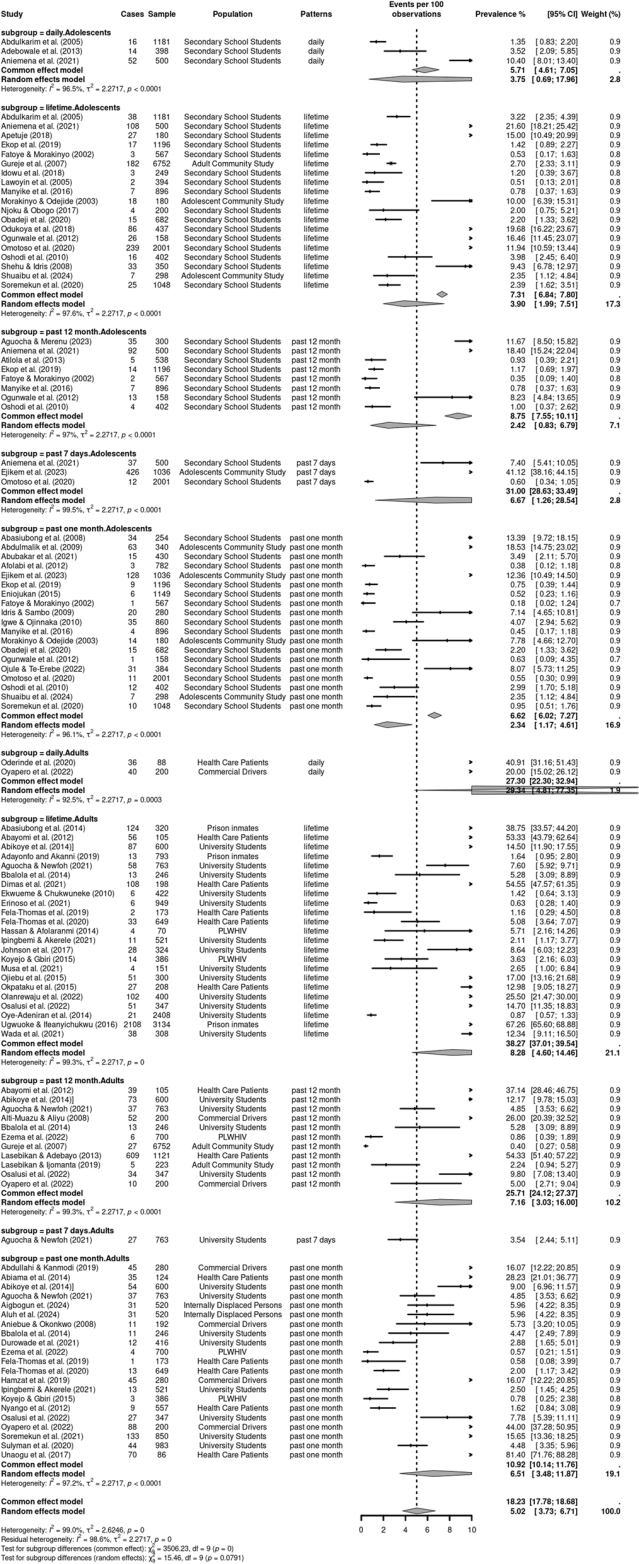
Forest plot of pooled prevalence estimates of cannabis use patterns stratified by population (adults and adolescents)

**Fig. 3:**
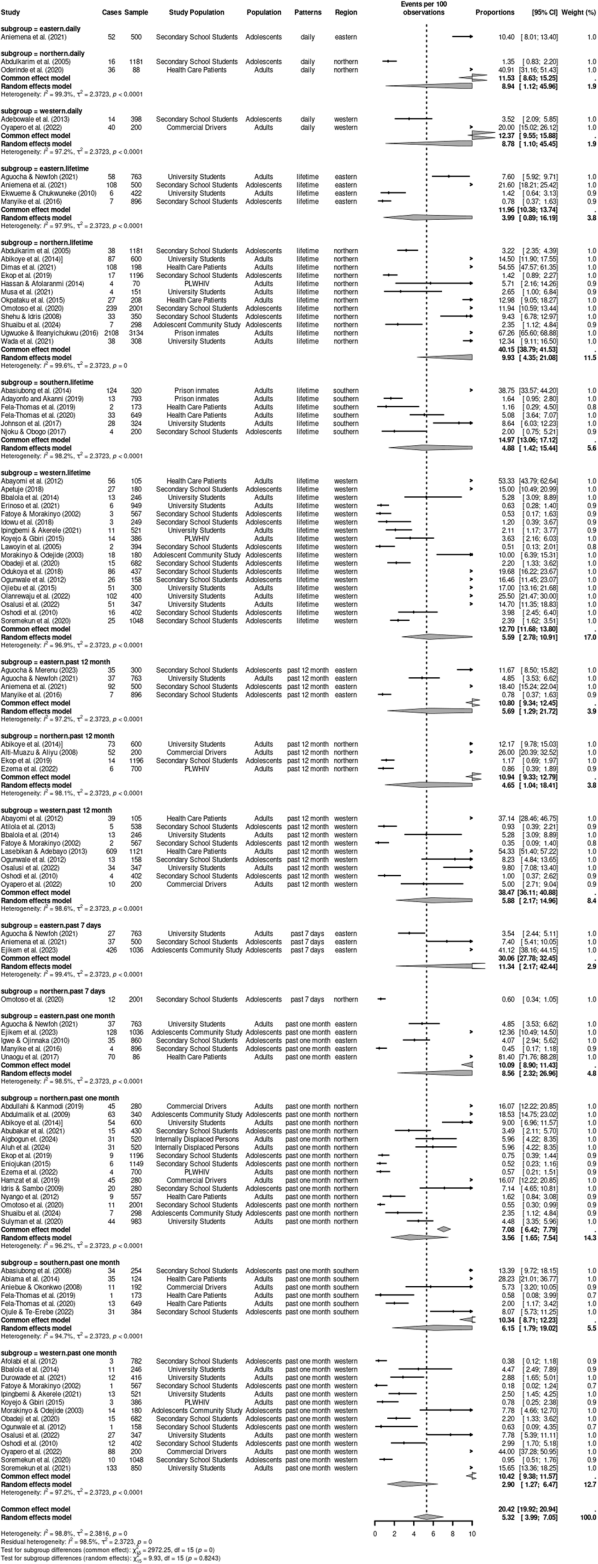
Forest plot of pooled prevalence estimates of cannabis use patterns stratified by region (western, northern, eastern and southern)

Participants’ age at first use of cannabis ranged from 10 years (Babalola 2014) or less to 11–14 years (Shehu 2008; Manyike et al. 2016). Figures 2 and 3 contain forest plots of the pooled prevalence estimates of patterns of cannabis use using a random effects model based on region and population (See Supplementary Table S4 for summary Table). The life-time prevalence of cannabis use ranged from 0.5 to 67.3%, with a pooled prevalence of 5.86% (95% CI: 3.46%–9.74). The past 12-month prevalence ranged from 0.4 to 54%, with a pooled prevalence of 4.54% (95% CI: 2.04%–9.83). The past one month prevalence ranged from 0.2 to 81.4%, with a pooled prevalence of 4.21%. The prevalence for past 7-day use was between 0.6 to 41% with a pooled prevalence of 5.66% (95% CI: 0.78%–31.36), while the prevalence for daily use was between 1.4 to 40.9%; this had the highest pooled prevalence at 12.21% (95% CI: 4.42%–29.50). However, the test of subgroup differences was not statistically significant (*Q* = 4.31, *df* = 4, *p* = 0.366), suggesting that when accounting for between study heterogeneity, the differences in the prevalence of cannabis use across the 5 patterns of use was not significant.

### Subgroup analyses of patterns of cannabis use based on population and region

We conducted subgroup analyses of patterns of use (e.g. lifetime, past 12 months, past one month, past 7 days and daily use) based on population (adolescents vs. adults) and pattern of use, using the random-effects model. Significant high heterogeneity was observed across the studies based on population (*I*^2^ = 99.0%, *p* < 0.001) and region (*I*^2^ = 98.98%, *p* < 0.001).

### Lifetime use

Lifetime use was examined by 42 studies. There was a significant statistical difference in the lifetime use prevalence across populations (*p* < 0.001) and regions *p*< 0.001). Adults had a significantly higher pooled prevalence of 8.28% (95% CI:4.60–14.46) compared to 3.90% (95% CI:1.99–7.51) among adolescents. The Northern region had a significantly higher lifetime prevalence of 9.93% (95% CI:4.36–21.08) than other regions. The highest lifetime prevalence among adults was 67.3%, reported in a study of 3134 prison inmates (95% CI: 65.60–68.88) in the Northern region (Ugwuoke 2016) while the highest prevalence among adolescents was 21.60% (95% CI: 18:21- 25.42), reported in a sample of 500 secondary school students (Aniemena et al. 2021).

### Past 12 months use

Nineteen studies examined past year cannabis use. The pooled prevalence estimate for past 12 months use among adults was 4.54% (95% CI: 2.04–9.83), which was significantly higher compared to adolescents, whose pooled prevalence estimate was 2.42% (95% CI: 8.75–6.7). The Western region had a significantly higher past month prevalence of 5.88% (95% CI: 2.78–10.91) exceeding other regions. Among adults, the highest past 12-month prevalence was 54.33%, reported in a study of 1121 trauma patients in the Western region (95% CI: 51:40–57:22) (Lasebikan 2013).

### Past one month use

Forty studies examined past one-month use of cannabis. The pooled prevalence estimate for past one month among adults was 6.51% (95% CI: 3.48–11.87), which was significantly higher compared to adolescents, whose pooled prevalence estimate was 2.34% (95% CI: 1.17–4.61). The highest prevalence of past one-month use among adults in a study of 88 adult Psychiatric patients and was 81.4% (95% CI:71.76–88.28) (Unaogu 2017). The highest prevalence of past one-month use among adolescents was 18.53% (CI; 14.75–23.02) in a sample of 340 Almarijis (children in informal religious schools) (Abdulmalik 2009). The eastern region had a significantly higher past one-month prevalence of 8.56% (95% CI: 2.32–26.96) compared to other regions.

### Past 7 days use

Four studies reported prevalence rates for past 7-days use. The pooled prevalence estimate for past 7-days use among adolescents was 6.67% (95% CI: 1.26–28.54), which was significantly higher compared to adults, whose pooled prevalence estimate was 3.54% (95% CI: 0.19–41.91). The highest prevalence of past 7 days use among adolescents was 41.2% (95% CI:38.16–44.15) in a sample of 1036 adolescents. The Eastern region had a significantly higher past 7 days prevalence of 11.34% (95% CI: 2.17–42.44) compared to the Northern regions. None of the reviewed studies were conducted in the Western and Southern regions.

### Daily use

Six studies examined daily cannabis use. Among adults, the pooled prevalence of daily use was 29.34% (95% CI: 4.81–77.34), higher than the 3.75% (95% CI:0.69–17.96) among adolescents. The highest prevalence of daily use among adolescents was 10.40% (95%CI; 8.01–13.40) in a sample of 500 secondary school students (Aniemena 2021). The Northern region had a significantly higher prevalence of daily cannabis use, 8.95% (95%CI:1.12–45.96), compared to the Eastern and Western regions.

#### Risk factors associated with the use of cannabis

Thirteen studies independently assessed the risk factors associated with the use of cannabis and reported qualitative data which varied across the studies (Supplementary Table S5). Being a male was a risk factor for cannabis use and was mostly reported among studies of secondary school students (Obadeji et al. 2020), university students (Babalola 2014), adults (Gureje et al. 2007), and primary health care patients (Fela-Thomas 2020). Other risk factors reported by studies conducted among university students included difficulty in studying (Abikoye 2014), being in the final year (usually 400 level), and being at a state university (Soremekun 2021), Additional risk factors reported in studies among secondary school students were age (Shehu 2008), being in a senior class (Soremekun 2020), father's cannabis use (Omotoso 2020), and negotiated unsupervised time (Odukoya 2018). One study examined the prevalence and pattern of psychoactive substance use among a sample of male children in an informal religious school (Almajiris) aged 5–16 years old (Abdulmalik 2009). Risk factors for cannabis use reported in the study were being older, being from a polygamous home, and parent separation.

#### The motivations for the use of cannabis

Only two studies independently assessed motivations for the use of cannabis. One study examined motivations for the use of Indian hemp among a sample of psychiatric patients (Oderinde 2020). Motivations reported in the study for the use of Indian hemp were to feel high (44.4%), compulsive urge (27.8%), to improve mood (11.1%), to prevent withdrawal syndrome (8.3%), to prolong the time of sexual intercourse (5.6%), and to relieve tiredness (2.8%). Motivations for the use of marijuana among secondary school students included relaxation, happiness, and to increase confidence (Shehu 2008).

#### Source of supply for marijuana

Only two studies independently assessed the sources of marijuana (Hamzat 2019; Afolabi 2012). Sources of marijuana included friends (50.7%) and peers, for commercial drivers and secondary school students, respectively. Additional sources of supply included vendors (53%) and stores/shops (36.8%) for commercial drivers (Hamzat 2019), and open drug market and village drug hawkers for secondary school students (Afolabi 2012).

### Discussion

Our review of 72 studies provides the pooled prevalence of cannabis use across different patterns of use (e.g. lifetime, past 12 months, past one month, past 7 days and daily use) based on regions (Eastern, Northern, Southern, and Western) and population (adolescents and adults) in Nigeria. The pooled prevalence estimates revealed notable variations in cannabis use patterns across populations and regions. Daily use was highest, followed by lifetime use, past 7 days, past 12 months, and past one month use. Prevalence was consistently higher among adult populations for lifetime, past 12 months, past one month and daily use compared to adolescents. Regionally, higher rates for lifetime use, past 12 months use, and past one month use were reported by studies from the Northern, Western and Eastern regions respectively.

To the best of our knowledge, this is the first systematic review and meta-analysis that has assessed the existing research literature examining the patterns, prevalence, and risk factors for cannabis use in Nigeria. The 72 studies included sampled a broad range of populations, including men, women, young people, healthcare patients, incarcerated persons, commercial transport workers, people living with HIV and AIDs (PLWHA), students and street children. The findings make important contributions to the current knowledge of cannabis use through summarizing the existing literature to reveal broad patterns and trends as well as generating ideas and hypotheses for further research.

A previous review examined the prevalence of cannabis use in Sub-Saharan Africa (Belete et al. 2023). The current study is an updated review of cannabis use in Nigeria and includes 72 studies conducted across the four regions of Nigeria compared to only 12 studies conducted in Western Nigeria that were included in the previous review (Belete et al. 2023). In the current study, the lifetime prevalence estimates for adolescents (4.05%) and adults (8.20%) are notably lower than the sub-Saharan regional averages of 7.9% and 12.6%, respectively (Belete et al. 2023). The past 12 months prevalence of cannabis use among adolescents is 2.42% (95% CI; 0.83–6.79), which is lower than the 5.2% (95% CI; 1.7–10.3) among adolescents in Sub-Saharan Africa (Belete et al. 2023). Social stigma and legal restrictions surrounding cannabis in Nigeria may lead to underreporting during surveys, particularly among younger populations. The pooled prevalence of past 12-months cannabis use among adults in this review is 7.16% (95% CI:3.03–15.99), which is high compared to the 2.2% in Sub-Saharan Africa (Belete et al. 2023) and the global average of 4% past 12-months use reported among adults in the 2024 World Drug Report (United 2024).

In this study, the prevalence of past 7-days use was 6.67% among adolescents (95% CI; 1.26–28.54), which was higher than among adults (3.5%, 95% CI; 0.19–41.91). The review also found a high prevalence of daily cannabis use among adults (29.34%; 95% CI; 4.81–77.34), a pattern of use that was not examined in the previous review. The highest prevalence of daily use was reported in the Northern (40.91%) and Western (20%) regions among a sample of health care patients (Oderinde 2020) and commercial drivers (Oyapero 2022), respectively. Comparatively, an estimated 1.3% of adults across the European Union engage in daily or near-daily cannabis use, with countries such as France and Germany recording above-average prevalence—particularly among individuals aged 15 to 34 (EMCDDA 2024). The high prevalence of daily cannabis use in Nigeria compared to the EU regions, reflects an interplay of environmental, socioeconomic, and psychosocial factors that sustain both its cultivation and consumption. Fertile rainforest zones in Western states like Ondo, Ekiti, and Ogun enable biannual harvesting, making cannabis both accessible and affordable (Nelson 2025; Nelson 2021b). Economic hardship and limited employment opportunities incentivize rural farmers to cultivate cannabis as a viable livelihood strategy (Dauda 2016). In urban centers in Western Nigeria such as Lagos and Ibadan, cannabis use is embedded within youth subcultures and informal economies, often serving as a coping mechanism for stress and trauma. Criminal sanctions fail to deter use, but rather exacerbate social and health harms through corrupt and extra-legal policing practices (Nelson 2025; Nelson 2021b).

The review found evidence of early initiation of cannabis consumption (10–14 years) much earlier than the average age of initiation in the national survey of drug use, which was estimated at 19 years (United 2019). This finding is troubling when viewed from a public health perspective. Early initiation of cannabis use is associated with an increased risk of negative social, health and behavioral outcomes, including other substance use and substance use disorders (Bagot 2015; Hall 2009b). It is also implicated in the development of learning difficulties and the early onset of psychotic disorders, especially in persons with pre-existing vulnerabilities and those who have greater severity of use (Bagot 2015; Hall 2009b). Thus, early cannabis use initiation, reported in these studies, is a trend that has negative public health implications. This trend is all the more concerning when cognizance is taken of the pattern of regular use (especially daily and nearly daily consumption) common among the adolescents and secondary school students in the reviewed literature.

The available evidence strongly suggests that cannabis use can have severe negative effects on some users, especially adolescents who initiate use early and young adults who become regular users (Hall 2009b). Thus, the cannabis use trends observed in the literature we have reviewed presents a strong case for interventions to prevent early initiation of cannabis use, including through both community and school-based programs such as the ‘Unplugged’ model that has already been piloted in some states (Vigna-Taglianti et al. 2021). This model has been demonstrated to prevent substance use initiation as well as progression to substance use disorders through the inculcation of personal and social skills.

The review also highlights an interesting trend characterized by high prevalence of use among adolescents and secondary school students (up to 30%) on the one hand, and low use prevalence among university students (4%) on the other. Although none of the studies we have reviewed used longitudinal designs, and therefore care should be taken not to interpret the findings longitudinally, when the prevalence of use among secondary school and university students (broadly suggesting adolescence and young adulthood) are juxtaposed, it could be hypothesized that the prevalence of use declines as people transition from adolescence to young adulthood or from secondary to tertiary level education. Studies have long reported declines in cannabis use during this period of transition, with some explaining it as a reflection of role transitions in early adulthood such as entering tertiary education (Dahl 2015). Nevertheless, this decline was only reported among university students and may not extend to less socially integrated young adults.

The prevalence of use was high among healthcare patients (e.g. psychiatric patients), commercial transport workers and incarcerated persons, which possibly included young adults. This prompts the question: do life-course transitions (in this case, tertiary education) act as a protective factor against problematic cannabis use in young adulthood? At a programmatic level, this indicates a need to improve access to substance use disorder treatment, including tailoring services to the needs of different groups (e.g. commercial transporters and incarcerated persons).

The studies reviewed here documented different sources of cannabis supply as well as a range of external factors and internal motivations influencing cannabis use, including gender (i.e. being male), academic difficulties, parental use, pleasure/relaxation, mood improvement, urge satisfaction and boosting self-confidence. Our findings reaffirm gender as a consistent predictor of cannabis use, aligning with global evidence that males engage in cannabis use more than females (United 2024). This gender disparity has also been documented in Nigerian university (Dumbili 2021; Ugwu 2024) and adult (United 2019) populations, where males report higher rates of cannabis and tramadol use. The association between parental substance use and adolescent cannabis use in our study corroborates prior Nigerian research showing that parental tobacco or alcohol use significantly increases the likelihood of adolescent cannabis initiation (Obisesan 2023).

Although the motives and risk factors reported in these studies are important predictors of cannabis use, they constitute individual/behavioral and proximal determinants of cannabis consumption. This implies that the macro-structural factors that shape substance use patterns and outcomes (as conceptualized, for instance, in the risk environment framework) (Rhodes 2002) are quantitatively under-studied in relation to cannabis use. By studying individual motives and behaviors in a largely decontextualized manner, current research could inadvertently reinforce the blameworthiness and stigmatization of problematic cannabis users. Recent studies that used qualitative methods to explore young people’s experiences of cannabis consumption have shown how the motivations and patterns of cannabis use are situated within wider contexts of poverty, structural violence and limited access to health and social services, which intersect to shape how, why and with what effects young people consume cannabis (Nelson 2021a; Nelson 2024; Nelson 2023). The structural patterning of cannabis use motivations offers a counterpoint to the stigmatization reinforced through existing quantitative findings that unduly responsibilize young people for drug-related harms, highlighting the importance of mixed-methods investigations to enable a more nuanced appreciation of the socioeconomic drivers of problematic cannabis use patterns.

Lifetime cannabis use, past 12 months and past one month use were notably highest in the Northern, Eastern and Western regions respectively, indicating a need for region-specific public health strategies. Additionally, although past one month use was higher among adults (6.51%) compared to adolescents (2.34%), the age of first initiation was between 10–14 years underscoring the importance of age-targeted interventions. To strengthen cannabis use prevention efforts in Nigeria, we recommend the following targeted policy options: expanding access to community-based treatment and harm reduction services, particularly in the northern, western and eastern regions, where there is a high prevalence of lifetime use, past 12 months and past one month use respectively; integrating evidence-based drug education into secondary school curricula, informed by the early age of initiation to cannabis use; and including modules on cannabis risks and coping strategies can help delay initiation and reduce stigma. Also, occupational screening and wellness programs need to be implemented for long-distance drivers, a group identified as high-risk, informed by the higher rates of daily cannabis use among this population. These targeted actions align with Nigeria’s National Drug Control Master Plan and reflect international best practices.

Furthermore, the review indicates directions for future research on cannabis use and associated harms. First, to balance the overwhelming focus on individual/proximal risk factors, it indicates a need to examine the associations between cannabis consumption patterns and associated social and health outcomes. Secondly, cannabis use prevalence and patterns also need to be examined through longitudinal, mixed-methods research, which aims to understand the role of life-course transitions in this decline. Such a research design could also help to identify the factors driving the escalation of cannabis use and the development of cannabis use disorders during young adulthood and adulthood. It could also help to determine if social integration (measured contextually by such indices as tertiary education, white collar employment etc.) protects against cannabis use disorders in young adulthood/adulthood. Finally, the review has identified a number of sources of cannabis supply, especially supply through vendors in rural settings, which demonstrates the need for research on the retail cannabis markets in rural Nigeria, an aspect of the illegal drug market that has received far less attention in emerging research, which is almost exclusively focused on urban settings (e.g Nelson 2024; Nelson 2023).

## Limitation

The study has some limitations. While the search strategy was designed to be comprehensive across multiple databases, one limitation is the omission of regionally specific or local terms for cannabis in the search strings. In Nigeria, cannabis is commonly referred to by various local names such as “marijuana” and “igbo,” as identified in several of the studies included. The absence of these terms may have led to the exclusion of relevant literature that relied solely on indigenous descriptors. Although this review captured and reported the terminology used within the studies themselves, future systematic reviews should consider incorporating culturally specific language into their search strategies to enhance the coverage and inclusivity of relevant evidence**.** Several studies in the review were cross-sectional with convenience samples thus limiting causal inference and generalizability. The pooled prevalence estimates included data from incarcerated individuals, psychiatric patients, and other institutional populations such as prison inmates and primary health care attendees. While these subgroups offer valuable insights into high risk-populations, their inclusion may limit generalizability, as they are not representative of the broader population. This may result in an upward bias when extrapolating prevalence rates to the general population. Future meta-analyses should conduct sub-group analysis to differentiate between the general and high-risk populations.

The lack of short-term cannabis use data from the western and southern regions limits the ability to assess recent consumption patterns and may skew regional comparisons. This absence could reflect underreporting, or limited research focus on these areas. Future studies in the regions should prioritize examining more recent consumption patterns to improve representativeness and inform targeted interventions.

## Conclusion

This review has examined the patterns, prevalence, risk factors and motivations for cannabis use among different social groups in Nigeria. In doing so, the review provides a useful summary of the existing research evidence on these topics, sheds light on troubling trends and their public health implications, and highlights directions for future research. Among other important findings, it reveals early initiation of cannabis use and overall high prevalence of consumption among several groups, including and especially young people, all of which points to the importance of investing in evidence-based prevention and treatment programs. The review also highlights the need for qualitative and mixed-methods studies to facilitate a deeper understanding of the contextual factors shaping motivations and patterns of cannabis use, thereby informing the development and tailoring of interventions.

## Supplementary Information

Supplementary Material 1

## Data Availability

All data analysed during this study are included in this published article [and its supplementary information files].
