# Supplementary material for: Cannabis use patterns, prevalence, and risk factors in Nigeria: a systematic review and meta-analysis
Source: J Cannabis Res. 2025 Nov 11;7:88. doi: 10.1186/s42238-025-00337-0 (PMC12606943; doi:10.1186/s42238-025-00337-0)
Supplement: Supplementary file 1 — Supplementary Material 1 [file 42238_2025_337_MOESM1_ESM.docx]

**Supplementary Tables for Cannabis Use Patterns, Prevalence and Risk Factors in Nigeria: A Systematic Review and Meta-analysis**

**Table of Content**

Page

Supplementary Table S1: Literature Search Strategy 2

Supplementary Table S2: Methodological quality and risk of bias assessment 6

Supplementary Table S3: Characteristics of studies included in the review 11

Supplementary Table S4: Patterns and prevalence of cannabis use stratified by

region and population 17

Supplementary Table S5: Summary of studies on risk factors and motivation for

cannabis use 19

# **Supplementary Table S1: Literature Search Strategy**

**DATABASE 1 – PubMed**

| **Search term** | **Search string** | **Search Results** |
| --- | --- | --- |
| #1 Substance use | ("substance use"[Title/Abstract] OR "drug use"[Title/Abstract] OR "substance misuse"[Title/Abstract] OR "substance abuse"[Title/Abstract] OR "substance dependence"[Title/Abstract] OR "substance use disorder"[Title/Abstract] OR "cannabis use"[Title/Abstract] OR "cannabis misuse"[Title/Abstract] OR "cannabis abuse"[Title/Abstract] OR "cannabis dependence"[Title/Abstract] OR "cannabis use disorder"[Title/Abstract]) | 136,765 |
| #2 Nigeria | ("Nigeria"[Title/Abstract]) | 39,468 |
| #3 Prevalence | ("prevalence"[Title/Abstract] OR "pattern"[Title/Abstract] OR "percent"[Title/Abstract] OR "proportion"[Title/Abstract] OR "practice"[Title/Abstract]) | 3,240,622 |
| 4#  Final search term | **#1 AND #2 AND #3**  (("substance use"[Title/Abstract] OR "drug use"[Title/Abstract] OR "substance misuse"[Title/Abstract] OR "substance abuse"[Title/Abstract] OR "substance dependence"[Title/Abstract] OR "substance use disorder"[Title/Abstract] OR "cannabis use"[Title/Abstract] OR "cannabis misuse"[Title/Abstract] OR "cannabis abuse"[Title/Abstract] OR "cannabis dependence"[Title/Abstract] OR "cannabis use disorder"[Title/Abstract]) AND "english"[Language] AND ("Nigeria"[Title/Abstract] AND "english"[Language]) AND (("prevalence"[Title/Abstract] OR "pattern"[Title/Abstract] OR "percent"[Title/Abstract] OR "proportion"[Title/Abstract] OR "practice"[Title/Abstract]) AND "english"[Language])) | 208 |
| #5  Filters | **Humans and From 2000/01/01 to 2023/12/31**  (("substance use"[Title/Abstract] OR "drug use"[Title/Abstract] OR "substance misuse"[Title/Abstract] OR "substance abuse"[Title/Abstract] OR "substance dependence"[Title/Abstract] OR "substance use disorder"[Title/Abstract] OR "cannabis use"[Title/Abstract] OR "cannabis misuse"[Title/Abstract] OR "cannabis abuse"[Title/Abstract] OR "cannabis dependence"[Title/Abstract] OR "cannabis use disorder"[Title/Abstract]) AND "english"[Language] AND ("Nigeria"[Title/Abstract] AND "english"[Language]) AND (("prevalence"[Title/Abstract] OR "pattern"[Title/Abstract] OR "percent"[Title/Abstract] OR "proportion"[Title/Abstract] OR "practice"[Title/Abstract]) AND "english"[Language])) AND ((2000/1/1:2022/12/31[pdat]) AND (english[Filter])) | 184 |

**DATABASE 2** - Web of Science

| **Search term** | **Search string** | **Search Results** |
| --- | --- | --- |
| #1 Substance use | ("substance use"[Title/Abstract] OR "drug use"[Title/Abstract] OR "substance misuse"[Title/Abstract] OR "substance abuse"[Title/Abstract] OR "substance dependence"[Title/Abstract] OR "substance use disorder"[Title/Abstract] OR "cannabis use"[Title/Abstract] OR "cannabis misuse"[Title/Abstract] OR "cannabis abuse"[Title/Abstract] OR "cannabis dependence"[Title/Abstract] OR "cannabis use disorder"[Title/Abstract]) | 1,126,957 |
| #2 Nigeria | ("Nigeria"[Title/Abstract]) | 40,948 |
| #3 Prevalence | ("prevalence"[Title/Abstract] OR "pattern"[Title/Abstract] OR "percent"[Title/Abstract] OR "proportion"[Title/Abstract] OR "practice"[Title/Abstract]) | 5,394 |
| 4#  Final search term | **#1 AND #2 AND #3**  (("substance use"[Title/Abstract] OR "drug use"[Title/Abstract] OR "substance misuse"[Title/Abstract] OR "substance abuse"[Title/Abstract] OR "substance dependence"[Title/Abstract] OR "substance use disorder"[Title/Abstract] OR "cannabis use"[Title/Abstract] OR "cannabis misuse"[Title/Abstract] OR "cannabis abuse"[Title/Abstract] OR "cannabis dependence"[Title/Abstract] OR "cannabis use disorder"[Title/Abstract]) AND "english"[Language] AND ("Nigeria"[Title/Abstract] AND "english"[Language]) AND (("prevalence"[Title/Abstract] OR "pattern"[Title/Abstract] OR "percent"[Title/Abstract] OR "proportion"[Title/Abstract] OR "practice"[Title/Abstract]) AND "english"[Language])) | 723 |
| #5  Filters | **From 2000/01/01 to 2022/12/31**  (("substance use"[Title/Abstract] OR "drug use"[Title/Abstract] OR "substance misuse"[Title/Abstract] OR "substance abuse"[Title/Abstract] OR "substance dependence"[Title/Abstract] OR "substance use disorder"[Title/Abstract] OR "cannabis use"[Title/Abstract] OR "cannabis misuse"[Title/Abstract] OR "cannabis abuse"[Title/Abstract] OR "cannabis dependence"[Title/Abstract] OR "cannabis use disorder"[Title/Abstract]) AND "english"[Language] AND ("Nigeria"[Title/Abstract] AND "english"[Language]) AND (("prevalence"[Title/Abstract] OR "pattern"[Title/Abstract] OR "percent"[Title/Abstract] OR "proportion"[Title/Abstract] OR "practice"[Title/Abstract]) AND "english"[Language])) AND ((2000/1/1:2022/12/31[pdat]) AND (english[Filter])) | 677 |

**DATABASE 3 – ProQuest**

| **Search term** | **Search string** | **Search Results** |
| --- | --- | --- |
| #1 Substance use | ("substance use" OR "drug use" OR "substance misuse" OR "substance abuse" OR "substance dependence" OR "substance use disorder" OR "cannabis use" OR "cannabis misuse" OR "cannabis abuse" OR "cannabis dependence" OR "cannabis use disorder") | 17,914 |
| #2 Nigeria | ("Nigeria") | 17,423 |
| #3 Prevalence | ("prevalence" OR "pattern" OR "percent" OR "proportion" OR "practice") | 707,219 |
| 4#  Final search term | **#1 AND #2 AND #3**  **Filters**  From January 2000 to December 2023 | 34 |

**DATABASE 4 -** EBSCO Host

APA PsycInfo, APA PsycArticles, CINAHL Plus with Full Text, and CINAHL Ultimate

| **Search term** | **Search string** | **Search Results** |
| --- | --- | --- |
| #1 Substance use | ("substance use" OR "drug use" OR "substance misuse" OR "substance abuse" OR "substance dependence" OR "substance use disorder" OR "cannabis use" OR "cannabis misuse" OR "cannabis abuse" OR "cannabis dependence" OR "cannabis use disorder") | 230,561 |
| #2 Nigeria | ("Nigeria") | 18,757 |
| #3 Prevalence | ("prevalence" OR "pattern" OR "percent" OR "proportion" OR "practice") | 2,317,614 |
| 4#  Final search term | **#1 AND #2 AND #3**  **Filters**  From January 2000 to December 2023 | 170 |

**DATABASE 5**: Google Scholar advanced search

| **Search term** | **Search string** | **Search Results** |
| --- | --- | --- |
| #1 Substance use | ("substance use" OR "drug use" OR "substance misuse" OR "substance abuse" OR "substance dependence" OR "substance use disorder" OR "cannabis use" OR "cannabis misuse" OR "cannabis abuse" OR "cannabis dependence" OR "cannabis use disorder") | 19700 |
| #2 Nigeria | ("Nigeria") | 612,000 |
| #3 Prevalence | ("prevalence" OR "pattern" OR "percent" OR "proportion" OR "practice") | 1,180,000 |
| 4#  Final search term | **#1 AND #2 AND #3**  **Filters**  From 2000 to 2023 | 70 |

**DATABASE 6 – African Journal Online (AJOL)**

| **Search term** | **Search string** | **Search Results** |
| --- | --- | --- |
| #1 Substance use | ("substance use" OR "drug use" OR "substance misuse" OR "substance abuse" OR "substance dependence" OR "substance use disorder" OR "cannabis use" OR "cannabis misuse" OR "cannabis abuse" OR "cannabis dependence" OR "cannabis use disorder") | 5870 |
| #2 Nigeria | ("Nigeria") | 14000 |
| #3 Prevalence | ("prevalence" OR "pattern" OR "percent" OR "proportion" OR "practice") | 169,000 |
| 4#  Final search term | **#1 AND #2 AND #3**  **Filters**  From 2000 to 2023 | 99 |

**Supplementary Table S2: Methodological quality and risk of bias assessment**

**Result of methodological quality assessment of all studies included in the review using the Joanna Briggs Institute (JBI)** Prevalence Critical Appraisal Tool

(Q1) Was the sample frame appropriate to address the target population?’;

(Q2) Were study participants recruited in an appropriate way?;

(Q3) Was the sample size adequate?;

(Q4) Were the study subjects and setting described in detail?;

(Q5) Was data analysis conducted with sufficient coverage of the identified sample?;

(Q6) Were valid methods used for the identification of the condition?;

(Q7) Was the condition measured in a standard, reliable way for all participants?;

(Q8) Was there appropriate statistical analysis?; and

(Q9) Was the response rate adequate, and if not, was the low response rate managed appropriately?.

| No. | Study Author (Year) | Q1 | Q2 | Q3 | Q4 | Q5 | Q6 | Q7 | Q8 | Q9 | Total score | Risk of Bias | Overall Appraisal |
| --- | --- | --- | --- | --- | --- | --- | --- | --- | --- | --- | --- | --- | --- |
| 1 | Abasiubong et al. (2014) | yes | unclear | yes | yes | yes | yes | unclear | yes | yes | 7 | Low | High Quality |
| 2 | Abasiubong et al. (2008) | yes | unclear | yes | yes | no | yes | unclear | no | no | 4 | Moderate | Moderate |
| 3 | Abayomi et al. (2012) | yes | no | no | no | yes | yes | yes | yes | yes | 6 | Low | Moderate Quality |
| 4 | Abdulkarim et al. (2005) | yes | yes | yes | yes | yes | yes | yes | yes | yes | 9 | Low | High Quality |
| 5 | Adayonfo and Akanni (2019) | yes | yes | yes | yes | yes | yes | unclear | yes | yes | 8 | Low | High Quality |
| 6 | Adebowale et al. (2013) | yes | yes | yes | yes | yes | yes | yes | yes | yes | 9 | Low | High Quality |
| 7 | Afolabi et al. (2012) | yes | unclear | yes | unclear | yes | yes | yes | yes | yes | 8 | Low | High Quality |
| 8 | Aguocha and Merenu (2023) | yes | yes | yes | yes | yes | yes | yes | yes | yes | 9 | Low | High Quality |
| 9 | Abdullahi Hamzat and Kehinde Kanmodi (2019) | unclear | unclear | yes | yes | yes | yes | unclear | yes | yes | 6 | Moderate | Moderate Quality |
| 10 | Abdulmalik et al. (2009) | unclear | yes | no | yes | yes | yes | unclear | yes | yes | 6 | Low | Moderate |
| 11 | Abiama et al. (2014) | yes | unclear | No | yes | yes | yes | yes | yes | yes | 7 | Low | High Quality |
| 12 | Abikoye et al. (2014) | yes | yes | yes | yes | yes | yes | yes | yes | yes | 9 | Low | High Quality |
| 13 | Aguocha and Nwefoh (2021) | yes | yes | yes | yes | yes | yes | yes | yes | yes | 9 | Low | High Quality |
| 14 | Aigbogun et al. (2024) | no | unclear | yes | unclear | yes | yes | yes | yes | yes | 6 | Moderate | Moderate Quality |
| 15 | Alti-Muazu and Aliyu (2008) | yes | yes | no | yes | yes | unclear | unclear | yes | yes | 6 | Moderate | Moderate Quality |
| 16 | Aluh et al. (2024) | no | unclear | yes | unclear | yes | yes | yes | yes | yes | 6 | Moderate | Moderate Quality |
| 17 | Aniebue and Okonkwo (2008) | no | unclear | no | unclear | yes | unclear | unclear | yes | unclear | 2 | High | Low Quality |
| 18 | Aniemena et al. (2021) | yes | unclear | yes | yes | yes | yes | yes | yes | yes | 8 | Low | High Quality |
| 19 | Apetuje Emmanuel Temidayo (2018) | yes | no | yes | yes | yes | unclear | unclear | unclear | yes | 5 | Moderate | Moderate Quality |
| 20 | Atilola et al. (2013) | yes | yes | yes | yes | yes | yes | yes | yes | yes | 9 | Low | High Quality |
| 21 | Babalola et al. (2014) | yes | unclear | yes | yes | yes | yes | yes | yes | yes | 8 | Low | High Quality |
| 22 | Dimas et al. (2021) | yes | yes | yes | yes | yes | yes | yes | yes | yes | 9 | Low | High Quality |
| 23 | Durowade et al. (2021) | yes | yes | yes | yes | yes | unclear | unclear | yes | yes | 7 | Low | High Quality |
| 24 | Ejikem et al. (2023) | yes | yes | yes | yes | yes | yes | unclear | yes | yes | 8 | Low | High Quality |
| 25 | Ekop et al. (2019) | yes | unclear | yes | yes | yes | unclear | unclear | yes | yes | 6 | Moderate | Moderate Quality |
| 26 | Ekwueme & Chukwuneke (2010) | yes | yes | yes | yes | yes | yes | unclear | yes | yes | 8 | Low | High Quality |
| 27 | Ipingbemi & Akerele (2021) | yes | yes | yes | yes | yes | yes | yes | yes | yes | 9 | Low | High Quality |
| 28 | Eniojukan (2015) | yes | yes | yes | yes | yes | unclear | unclear | yes | yes | 7 | Low | High Quality |
| 29 | Erinoso et al. (2021) | yes | yes | yes | yes | yes | yes | yes | yes | yes | 9 | Low | High Quality |
| 30 | Ezema et al. (2022) | yes | yes | yes | yes | yes | yes | yes | yes | yes | 9 | Low | High Quality |
| 31 | Fatoye & Morakinyo (2002) | yes | yes | yes | yes | yes | yes | yes | yes | yes | 9 | Low | High Quality |
| 32 | Fela-Thomas et al. (2020) | yes | yes | yes | yes | yes | yes | yes | yes | yes | 9 | Low | High Quality |
| 33 | Fela-Thomas et al. (2019) | yes | yes | no | yes | yes | yes | yes | yes | yes | 8 | Low | High Quality |
| 34 | Gureje et al. (2007) | yes | yes | yes | yes | yes | yes | yes | yes | yes | 9 | Low | High Quality |
| 35 | Hamzat et al. (2019) | yes | yes | no | yes | yes | yes | no | yes | yes | 7 | Low | High Quality |
| 36 | Hassan & Afolaranmi (2014) | yes | yes | no | yes | yes | yes | no | yes | yes | 7 | Low | High Quality |
| 37 | Idowu et al. (2018) | yes | yes | no | yes | yes | yes | no | yes | yes | 7 | Low | High Quality |
| 38 | Idris & Sambo (2009) | yes | yes | no | yes | yes | yes | no | yes | yes | 7 | Low | High Quality |
| 39 | Igwe & Ojinnaka (2010) | yes | yes | yes | yes | yes | yes | yes | yes | yes | 9 | Low | High Quality |
| 40 | Oderinde et al. (2020) | yes | unclear | no | yes | yes | yes | unclear | yes | yes | 6 | Moderate | Moderate Quality |
| 41 | Koyejo & Gbiri (2015) | yes | unclear | yes | yes | yes | yes | yes | yes | yes | 8 | Low | High Quality |
| 42 | Lasebikan & Adebayo (2013) | yes | yes | yes | yes | yes | yes | yes | yes | yes | 9 | Low | High Quality |
| 43 | Lasebikan & Ijomanta (2019) | yes | unclear | yes | yes | yes | yes | yes | yes | yes | 8 | Low | High Quality |
| 44 | Lawoyin et al. (2005) | yes | yes | yes | yes | yes | unclear | unclear | yes | yes | 7 | Low | High Quality |
| 45 | Manyike et al. (2016) | yes | yes | yes | yes | yes | yes | yes | yes | yes | 9 | Low | High Quality |
| 46 | Morakinyo & Odejide (2003) | unclear | yes | no | yes | yes | yes | yes | yes | yes | 7 | Low | High Quality |
| 47 | Musa et al. (2021) | yes | yes | no | yes | yes | yes | unclear | no | yes | 6 | Moderate | Moderate Quality |
| 48 | Njoku & Obogo (2017) | yes | unclear | no | no | yes | yes | yes | yes | yes | 7 | Low | High Quality |
| 49 | Nyango et al. (2012) | yes | yes | yes | yes | yes | yes | unclear | yes | yes | 8 | Low | High Quality |
| 50 | Obadeji et al. (2020) | yes | yes | yes | yes | yes | yes | unclear | yes | yes | 8 | Low | High Quality |
| 51 | Oderinde et al. (2020) | yes | unclear | no | yes | yes | yes | unclear | yes | yes | 6 | Moderate | Moderate Quality |
| 52 | Odukoya et al. (2018) | yes | yes | yes | yes | yes | yes | yes | yes | yes | 9 | Low | High Quality |
| 53 | Johnson et al. (2017) | yes | unclear | no | yes | yes | yes | unclear | yes | yes | 6 | Low | High Quality |
| 54 | Ojieabu et al. (2015) | yes | yes | yes | yes | yes | yes | unclear | yes | yes | 8 | Low | High Quality |
| 55 | Ojule & Te-Erebe (2022) | yes | yes | yes | yes | yes | yes | unclear | yes | yes | 8 | Low | High Quality |
| 56 | Okpataku et al. (2015) | yes | yes | yes | yes | yes | yes | yes | yes | yes | 9 | Low | High Quality |
| 57 | Olanrewaju et al. (2022) | yes | yes | yes | yes | yes | yes | yes | yes | yes | 9 | Low | High Quality |
| 58 | Ogunwale et al. (2012) | yes | yes | no | yes | yes | yes | yes | yes | yes | 8 | Low | High Quality |
| 59 | Omotoso et al. (2020) | yes | yes | yes | yes | yes | yes | yes | yes | yes | 9 | Low | High Quality |
| 60 | Osalusi et al. (2022) | yes | yes | yes | yes | yes | yes | yes | yes | yes | 9 | Low | High Quality |
| 61 | Oshodi et al. (2010) | yes | yes | yes | yes | yes | yes | yes | yes | yes | 9 | Low | High Quality |
| 62 | Oyapero et al. (2022) | yes | yes | yes | yes | yes | yes | yes | yes | yes | 9 | Low | High Quality |
| 63 | Oye-Adeniran et al. (2014) | yes | yes | yes | yes | yes | yes | unclear | yes | yes | 8 | Low | High Quality |
| 64 | Shehu & Idris (2008) | yes | yes | yes | yes | yes | yes | unclear | yes | yes | 8 | Low | High Quality |
| 65 | Shuaibu et al. (2024) | yes | yes | yes | yes | yes | yes | yes | yes | yes | 9 | Low | High Quality |
| 66 | Soremekun et al. (2020) | yes | yes | yes | yes | yes | yes | yes | yes | yes | 9 | Low | High Quality |
| 67 | Soremekun et al. (2021) | yes | yes | yes | yes | yes | yes | yes | yes | yes | 9 | Low | High Quality |
| 68 | Sulyman et al. (2020) | yes | yes | yes | yes | yes | yes | yes | yes | yes | 9 | Low | High Quality |
| 69 | Ugwuoke & Ifeanyichukwu (2016) | yes | unclear | yes | yes | yes | yes | unclear | no | yes | 6 | Moderate | Moderate Quality |
| 70 | Unaogu et al. (2017) | yes | unclear | no | yes | yes | yes | unclear | yes | yes | 7 | Low | High Quality |
| 71 | Abubakar et al. (2021) | yes | yes | yes | yes | yes | yes | unclear | yes | yes | 8 | Low | High Quality |
| 72 | Wada et al. (2021) | yes | yes | yes | yes | yes | yes | unclear | yes | yes | 8 | Low | High Quality |

**Supplementary Table S3: Characteristics of studies included in the review**

| **Study** | **Region** | **n** | **Males n (%)** | **Females n (%)** | **Response Rate (%)** | **Age Range** | **Mean Age (SD)** | **Population** | **Patterns and prevalence of Cannabis Use** | | | | |
| --- | --- | --- | --- | --- | --- | --- | --- | --- | --- | --- | --- | --- | --- |
|  |  |  |  |  |  |  |  |  | **Daily Use (n)** | **Past 7 Days Use (n)** | **Past One month (n)** | **Past 12 Months (n)** | **Lifetime (n)** |
| Abasiubong et al. (2014) | Southern | 320 | 320 (100) |  | 94.7 |  | 23.7 (3.9) | Prison Inmates | - | - | - | - | 38.8% (124) |
| Abasiubong et al. (2008) | Southern | 254 | 160 (63) | 94 (37 | 63.5 |  | 17.1 (2.0) | Secondary School Students | - | - | 13.4% (34) | - | - |
| Abayomi et al. (2012) | Western | 105 | 90 (85.7 | 15 (14.3) | 94.6 |  | 31 (8.6) | Health Care Patients | - | - | - | 37.1% (39) | 53.3% (56) |
| Abdulkarim et al. (2005) | Northern | 1181 | 608 (51.9) | 563 (48.1) | 98.4 | 10-19 |  | Secondary School Students | 1.4% (16) | - | - | - | 3.4% (38) |
| Adayonfo and Akanni (2019) | Southern | 793 | 775 (97.7) | 18 (2.3) | 98.2 | 17-67 | 33 | Prison Inmates | - | - | - | - | 18.6% (13) |
| Adebowale et al. (2013) | Western | 398 | 180 (45.2) | 218 (54.8) | 95 |  | 13.8 (1.9) | Secondary School Students | 3.5% (14) | - | - | - | - |
| Afolabi et al. (2012) | Western | 782 | 379 (48.5) | 403 (51.5) | 98 |  |  | Secondary School Students | - | - | 1.1% (3) | - | - |
| Aguocha and Merenu (2023) | Eastern | 300 | 212 (70.7) | 88 (29.3) | 92.5 |  | 15.04 (1.68) | Secondary School Students | - | - | - | 11.7% (35) | - |
| Abdullahi Hamzat and Kehinde Kanmodi (2019) | Northern | 280 | 280 (100) |  | 96.9 |  | 40.63 (10.09) | Commercial Drivers | - | - | 16.1% (45) | - | - |
| Abdulmalik et al. (2009) | Northern | 340 | 340 (100) |  | 98 | 5-16 | 11.2 (3) | Almajiris | - | - | 18.5% (63) | - | - |
| Abiama et al. (2014) | Southern | 124 | 77 (62.1) | 47 (37.9) | 100 | 18-52 | 32.72 | Psychiatric Patients | - | - | 28.3% (35) | - | - |
| Abikoye et al. (2014) | Northern | 600 | 295 (49.2) | 305 (50.8) | 96.2 | 18 - 41 |  | University Students | - | - | 9% (54) | 12.2% (73) | 14.5% (87) |
| Aguocha and Nwefoh (2021) | Eastern | 763 | 323 (42.3) | 440 (57.7) | 92.3 | 18-30 |  | University Students | - | 3.5% (27) | 4.8% (37) | 4.8% (37) | 7.6% (58) |
| Aigbogun et al. (2024) | Northern | 520 | 124 (23.8) | 396 (76.2) | 86.7 |  | NA | Internally Displaced Persons | - | - | 5.9% (31) | - | - |
| Alti-Muazu and Aliyu (2008) | Northern | 200 | 200 (100) |  | 100 |  | 25.4 (3.9) | Commercial Drivers | - | - | - | 25.8% (52) | - |
| Aluh et al. (2024) | Northern | 520 | 124 (23.8) | 396 (76.2) | 86.7 |  | NA | Internally Displaced Persons | - | - | 5.9% (31) | - | - |
| Aniebue and Okonkwo (2008) | Southern | 192 | 192 (100) |  | not reported |  | 37.9 (9.1) | Commercial Drivers | - | - | 3.2% (11) | - | - |
| Aniemena et al. (2021) | Eastern | 500 | 337 (67.4) | 163 (32.6) | 100 | 10-19 |  | Secondary School Students | 10.4% (52) | 7.4% (37) | - | 18.4% (92) | 21.6% (108) |
| Apetuje (2018) | Western | 180 | 99 (55) | 81 (45) | 100 | 12 - 44 |  | Secondary School Students | - | - | - | - | 14.6% (26) |
| Atilola et al. (2013) | Western | 538 | 244 (44.4) | 294 (55.6) | 92 |  | 15.1 (1.4) | Secondary School Students | - | - | - | 1% (5) | - |
| Babalola et al. (2014) | Western | 246 | 130 (52.8) | 116 (47.7) | 88.5 |  | 26.8 | University Students | - | - | 4.5% (11) | 5.3 % (13) | 5.3% (13) |
| Dimas et al. (2021) | Northern | 198 | 140 (70.7) | 58 (29.3) | 100 |  |  | Substance Users | - | - | - | - | 54.5% (108) |
| Durowade et al. (2021) | Western | 416 | 188 (45.2) | 228 (54.8) | 96.7 |  |  | University Students | - | - | 4% (12) | - |  |
| Ejikem et al. (2023) | Eastern | 1036 |  |  | 95 | 10-19 |  | Adolescents | - | 41.10% | 53.5% (554) | - | - |
| Ekop et al. (2019) | Northern | 1196 | 555 (46.4%) | 641 (53.6) | 100 |  |  | Adolescents | - | - | 0.8% (9) | 1.2% (14) | 1.4% (17) |
| Ekwueme & Chukwuneke (2010) | Eastern | 422 |  |  | 100 |  |  | University Students | - | - | - | - | 1.4% (6) |
| Ipingbemi & Akerele (2021) | Western | 521 | 252 (48.4) | 269 (51.6) | 85.7 |  | 20.5 (2.4) | University Students | - | - | 2.5% (13) | - | 2.1% (11) |
| Eniojukan (2015) | Northern | 1149 | 662 (57.7) | 487 (42.5) | 91.9 |  |  | Secondary School Students | - | - | 8.3% (6) | - | - |
| Erinoso et al. (2021) | Western | 949 | 421 (44.4) | 528 (55.6) | 94.3 | 15 - 35 | 23.26 (3.97) | Secondary School Students | - | - | - | - | 8.1% (6) |
| Ezema et al. (2022) | Northern | 700 | 259 (37) | 441 (63) | 100 |  | 39.3 (10.1) | People Living with HIV | - | - | 0.6% (4) | 0.9% (6) | - |
| Fatoye & Morakinyo (2002) | Western | 567 | 266 (47.3) | 276 (52.7) | 93.7 |  | 17 (1.69) | Secondary School Students | - | - | 0.2% (1) | 0.4% (2) | 0.5% (3) |
| Fela-Thomas et al. (2020) | Southern | 649 | 371 (57.2) | 277 (42.7) | 99.8 | 14-59 Years | 37.5 (11.55) | Health Care Patients | - | - | 2.0% (13) | - | 5.1% (33) |
| Fela-Thomas et al. (2019) | Southern | 173 | 90 (52) | 83 (48) | 100 | 60 -89 Years |  | Health Care Patients | - | - | 0.6% (1) | - | 1.2% (2) |
| Gureje et al. (2007) | All Regions | 6752 | 3307 (69.4) | 3445 (46.5) | 79 | 18-65 |  | Adults | - | - | - | 0.4% (27) | 2.7% (182) |
| Hamzat et al. (2019) | Northern | 280 | 280 (100) |  | 96.9 |  | 40.63 (10.09) | Commercial Drivers | - | - | 16.1% (45) | - | - |
| Hassan & Afolaranmi (2014) | Northern | 70 | 37 (52.9) | 33 (47.1) | 100 | 18-49 | 30 (2.03) | People Living with HIV | - | - | - | - | 5.7% (4) |
| Idowu et al. (2018) | Western | 249 | 127 (51) | 122 (49) | 90 |  | 16.3 (2) | Secondary School Students | - | - | - | - | 1% (3) |
| Idris & Sambo (2009) | Northern | 280 | 195 (69.64) | 85 (30.36) | 100 | ≥ 13 |  | Secondary School Students | - | - | 7.1% (20) | - | - |
| Igwe & Ojinnaka (2010) | Eastern | 860 | 499 (57.4) | 360 (42.6) | 95.6 | 10-19 | 16.9 (1.7) | Adolescents | - | - | 4.1% (35) | - | - |
| Oderinde et al. (2020) | Northern | 88 | 85 (96.6) | 3 (3.4) | 100 | 10 - ≥ 50 |  | Psychiatric Patients | 40.9% (36) |  | - | - | - |
| Koyejo & Gbiri (2015) | Western | 386 | 129 (33.51) | 256 (66.49) | 100 | 10 - ≥ 50 | 37.62 (9.16) | People Living with HIV | - | - | 0.8% (3) | - | 3.6% (14) |
| Lasebikan & Adebayo (2013) | Western | 1121 | 594 (88.1) | 526 (11.6) | 82.5 |  |  | Trauma Patients | - | - | - | 54% (609) | - |
| Lasebikan & Ijomanta (2019) | NA | 223 |  |  | 100 | 24-58 | 38 | Military Officers | - | - | - | 33.3% (5) | - |
| Lawoyin et al. (2005) | Western | 394 | 217 (55.1) | 177 (44.9) | 101 | 14-24 |  | Secondary School Students | - | - | - | - | 0.7% (2) |
| Manyike et al. (2016) | Eastern | 896 |  |  | 99.6 | 15-19 | 15.9 (1.04) | Secondary School Students | - | - | 0.4% (4) | 0.8% (7) | 0.8% (7) |
| Morakinyo & Odejide (2003) | Western | 180 | 174 (96.7) | 6 (3.3) | 100 | 8-18 | 14.6 (2.6) | Street Children | - | - | 7.8% (14) | - | 10% |
| Musa et al. (2021) | Northern | 151 | 97 (64.2) | 54 (35.8) | 100 |  | 27.3 (6.2) | University Students | - | - | - | - | 14.3% (4) |
| Njoku & Obogo (2017) | Southern | 200 | - | - | 95 |  |  | Secondary School Students | - | - | - | - | 10% (4) |
| Nyango et al. (2012) | Northern | 557 | 557 (100) |  | 100 | 15-48 | 29.2 (5.3) | Pregnant Women | - | - | 1.6% (9) | - | - |
| Obadeji et al. (2020) | Western | 682 | 391 (57.3) | 291 (42.7) | 98.2 | 13-19 | 15.75 (1.35) | Secondary School Students | - | - | 2.2% (15) | - | 2.2% (15) |
| Oderinde et al. (2020) | Northern | 88 | 85 (96.6) | 3 (3.4) | 100 |  |  | Psychiatric Patients | 40.9% (36) | - | - | - | - |
| Odukoya et al. (2018) | Western | 437 | 204 (46.7) | 233 (53.3) | 100 | 10-19 | 15.30 (1.60) | Secondary School Students | - | - | - | - | 19.7% (86) |
| Johnson et al. (2017) | Southern | 324 | 170 (52.5) | 154 (47.5) | 92 | 18-25 | 21.57 (1.96) | University Students | - | - | - | - | 31% (28) |
| Ojieabu et al. (2015) | Western | 300 | 149 (49.7) | 151 (50.3) | 100 | 15-50 |  | University Students | - | - | - | - | 17% (51) |
| Ojule & Te-Erebe (2022) | Southern | 384 | 195 (50.8) | 189 (49.2) | 100 | 10-25 |  | Secondary School Students | - | - | 8.1% (31) | - | - |
| Okpataku et al. (2015) | Northern | 208 | 110 (52/9) | 98 (47.1) | 100 |  | 36.72 (12.69) | Psychiatric Patients | - | - | - | - | 13% (27) |
| Olanrewaju et al. (2022) | Western | 400 | 128 (32) | 272 (68) | 83.3 | 15-24 |  | University Students | - | - | - | - | 25.5% (102) |
| Ogunwale et al. (2012) | Western | 54 |  |  | 100 |  |  | Adolescents | - | - | 1.9% (1) | 24.1% (13) | 28.1% (26) |
| Omotoso et al. (2020) | Northern | 2001 | 1083 (54.1) | 918 (45.9) | 92.3 |  | 15.05 (2.24) | Secondary School Students | - | 0.6% (12) | - | - | 11.9% (239) |
| Osalusi et al. (2022) | Western | 347 | 270 (69.7) | 117 (30.23 | 96.8 | 14-30 | 20.51 (2.91) | University Students | - | - | 6.9% (27) | 8.8% (34) | 13.2% (51) |
| Oshodi et al. (2010) | Western | 402 | 175 (43.5) | 227 (56.5) | 100 | 11-20 | 15.9 | Secondary School Students | - | - | 3.3% (12) | 1.1% (4) | 4.4% (16) |
| Oyapero et al. (2022) | Western | 200 | 193 (96.5) | 7 (3.5) | 100 |  | 42.70 (10.5) | Commercial Drivers | 20% (40) | - | 44% (88) | 5% (10) | - |
| Oye-Adeniran et al. (2014) | All Regions | 2408 | 2408 (100) |  | 100 | 17-49 | 21.6 (2.9) | University Students | - | - | - | - | 1% (21) |
| Shehu & Idris (2008) | Northern | 350 | 262 (74.9) | 88 (25.1) | 97.2 |  |  | Secondary School Students | - | - | - | - | 9.4% (33) |
| Shuaibu et al. (2024) | Northern | 298 | 112 (37.6) | 186 (62.1) | 100 | 10-19 | 13.5 (2.3) | Adolescents | - | - | 2.3% (7) | - | 2.3% (7) |
| Soremekun et al. (2020) | Western | 1048 | 533 (50.86) | 515 (49.14) | 100 |  |  | Secondary School Students | - | - | 1% (10) | - | 2.4% (25) |
| Soremekun et al. (2021) | Western | 850 |  |  | 97.2 |  |  | University Students | - | - | 15.7% (133) | - | - |
| Sulyman et al. (2020) | Northern | 983 | 560 (57) | 423 (43) | 93.9 | 17-38 | 23.3 (3.4) | University Students | - |  | 4.5% (44) | - | - |
| Ugwuoke & Ifeanyichukwu (2016) | Northern | 3134 | 3112 (99.3) | 22 (0.7) | 100 | 19-60 |  | Prison Inmates | - | - | - | - | 67.3% (2108) |
| Unaogu et al. (2017) | Eastern | 86 | 83 (96.5) | 3 (3.5) | 100 |  | 30.88 (8.49) | Psychiatric Patients | - |  | 81.4% (70) | - | - |
| Abubakar et al. (2021) | Northern | 430 | 284 (68.4) | 146 (31.6) | 100 |  | 16.3 (3.1) | Secondary School Students | - | - | 21.4% (15) | - | - |

**Supplementary Table S4: Patterns and prevalence of cannabis use stratified by region and population**

|  |  | **Lifetime CU** | **Past 12 months CU** | **Past One Month CU** | **Past 7 days CU** | **Daily CU** |
| --- | --- | --- | --- | --- | --- | --- |
| **Total** | *k* | 42 | 19 | 40 | 4 | 6 |
|  | Pooled prevalence | 5.86% | 4.54% | 4.21% | 5.66% | 12.21% |
|  | 95% CI | (3.46 - 9.74) | (2.04 – 9.83) | (2.92 – 6.02) | (0.78 – 31.36) | (4.42 – 29.50) |
|  | *I*^2^ | 99.35% | 99% | 96.9% | 99.4% | 96.9% |
|  | Q | 5879.96 | 1803.84 | 1249.08 | 522.88 | 1249.08 |
|  | *p* | *p*=0 | *p*=0 | *p<*0.0001 | *p<*0.0001 | *p<*0.0001 |
| **Region** |  |  |  |  |  |  |
| **Eastern** | *k* | 4 | 4 | 5 | 3 | 1 |
|  | Pooled prevalence | 3.99% | 5.69% | 8.56% | 11.34% | 10.40% |
|  | 95% CI | (0.89 – 16.19) | (1.29 – 21.72) | (2.32 – 26.96) | (2.17 – 42.22) | (0.56 – 70.66) |
|  | *I*^2^ | 97.9% | 97.2% | 98.6% | 99.4% | - |
|  | Q | 141.90 | 107.50 | 275.59 | 313.81 | - |
|  | *p* | *p<*0.0001 | *p<*0.0001 | *p<*0.0001 | *p<*0.0001 |  |
| **Northern** | *k* | 12 | 4 | 15 | 1 | 1 |
|  | Pooled prevalence | 9.93% | 4.65% | 3.56% | 0.60% | 8.94% |
|  | 95% CI | (4.36 – 21.08) | (1.04 – 18.41) | (1.65 – 7.54) | (0.03 – 11.52) | (1.12 – 45.96) |
|  | *I*^2^ | 99.6% | 98.1% | 96.2% | 99.3% | 99.3% |
|  | Q | 2514.35 | 161.02 | 370.91 | 139.24 | 139.24 |
|  | *p* | 0 | *p<*0.0001 | *p<*0.0001 |  |  |
| **Southern** | *n*= | 6 | - | 6 | - | - |
|  | Pooled prevalence | 4.88% |  | 6.15% |  |  |
|  | 95% CI | (1.42 – 15.44) |  | (1.79 – 19.02) |  |  |
|  | *I*^2^ | 98.2% |  | 94.7% |  |  |
|  | Q | 282.27 |  | 93.71 |  |  |
|  | *p* | *p<*0.0001 |  | *p<*0.0001 |  |  |
| **Western** | *k* | 18 | 9 | 14 | - | 2 |
|  | Pooled prevalence | 5.59% | 5.88% | 2.90% |  | 8.78% |
|  | 95% CI | (2.78 – 10.91) | (21.17 – 14.96) | (1.27 – 6.47) |  | (1.10 – 45.45) |
|  | *I*^2^ | 96.9% | 98.6% | 97.2% |  | 97.2% |
|  | Q | 555.49 | 592.43 | 471.57 |  | 35.21 |
|  | *p* | *p<*0.0001 | *p<*0.0001 | *p<*0.0001 |  | *p<*0.0001 |
| **Population** |  |  |  |  |  |  |
| **Adolescents** | *k* | 19 | 8 | 19 | 3 | 3 |
|  | Pooled prevalence | 3.90% | 2.42% | 2.34% | 6.67% | 3.75% |
|  | 95% CI | (1.99 – 7.51) | (0.83 – 6.79) | (1.17 – 4.61) | (1.26 – 28.54) | (0.69 – 17.96) |
|  | *I*^2^ | 97.6% | 97% | 96.1% | 99.5% | 96.5% |
|  | Q | 753.68 | 230.63 | 464.14 | 372.57 | 57.95 |
|  | *p* | *p<*0.0001 | *p<*0.0001 | *p<*0.0001 | *p<*0.0001 | *p<*0.0001 |
| **Adult** | *k* | 23 | 11 | 21 | 1 | 2 |
|  | Pooled prevalence | 8.28% | 7.16% | 6.51% | 3.54% | 29.34% |
|  | 95% CI | (4.60 – 14.46) | (3.03 – 15.99) | (3.48 – 11.87) | (0.19 – 41.91) | (4.81 – 77.34) |
|  | *I*^2^ | 99.3% | 99.3% | 97.2% |  | 92.5% |
|  | Q | 3055.91 | 1380.91 | 717.04 |  | 13.26 |
|  | *p* | *p=0* | *p<*0.0001 | *p<*0.0001 |  | *p*=0.0003 |

Abbreviations: *k* = number of studies; CU = Cannabis Use; CI = confidence interval.

**Supplementary Table S5: Summary of studies on risk factors and motivation for cannabis use**

| **Author (Year)** | **Study Aims** | | **Sample Description**  **(Population/Sample size/Age)** | | **City, State/Region** | | **Measures/**  **Other substances studied** | | **Term adopted for cannabis** | | **Results** | | | | | |
| --- | --- | --- | --- | --- | --- | --- | --- | --- | --- | --- | --- | --- | --- | --- | --- | --- |
|  |  |  |  |  |  |  |  |  |  |  | **Prevalence of cannabis use** | | **Risk factors for cannabis use** | | **Motivation for use of Cannabis** | |
| Abasiubong et al. (2014) | The study compared the pattern of substance use in two cities. | | Prison Inmates  N = 320 (Males only)  Uyo (n = 179)  Kiru (n = 141)  *Mean age* Uyo = 23.7 ± 3.9 years | | Uyo and Kiru, AkwaIbom State/Southern | | WHO Students substance use survey-modified/Yes | | Indian hemp | | Uyo (n = 63, 35.2%)  Kiru (n = 61, 43.3%) | | NA | | Motivation for substance use was studied, so findings are not specific to cannabis use. | |
| Abasiubong et al. (2008) | To determine the prevalence of substance use amongst  secondary school students | | SS1 – SS3 Secondary school students, N = 254  Uyo (n = 119),  Males = 76; Females = 43  *Mean age* = 17.1 ±2.0 years  Eket (n = 135)  Males = 84; Females = 51  *Mean age* = 16.6 ±1.7 years | | Uyo and Eket, AkwaIbom State/Southern | | WHO Students substance use survey-modified/Yes | | Indian hemp | | Uyo (n = 13, 10.9%)  Eket (n = 21, 15.6%)  Only males used Indian hemp. The use of Indian hemp was found in higher classes. | | Parents marital status | | NA | |
| Abayomi et al. (2012) | To determine the prevalence and correlates of substance use among persons with mental disorders. | | Psychiatric patients N = 105  Males = 90 (85.7%)  Females = 15 (14.3%)  19 – 56 Years  *Mean age* = 31± 8.6 years | | Aro, Ogun State/Western | | ASSIST/Yes | | Cannabis | | lifetime use = 56 (53.3%)  Hazardous and past 3 months use = 39 (37.1%) | | Studied but not specific to cannabis use. | | NA | |
| Abdulkarim et al. (2005) | To document the  types and frequency of drug use | | Secondary school students  N = 1181  Males = 608 (51.9%)  Females 563 (48.1%)  10 – 19 years | | Ilorin, Kwara State/Northern | | WHO Students substance use survey-modified/Yes | | Cannabis | | lifetime use = 38 (3.4%)  daily use = 16 (1.4%) | | Use of cigarettes and cocaine, respectively | | NA | |
| Adayonfo and Akanni (2019) | To examine the prevalence of sexual offense | | Prison inmates  N = 793  Males = 775  Females = 18  Age 17 – 67 years  *Mean age = 33 years* | | Benin city, Edo State/Southern | | WHO Student Drug Use Questionnaire/Yes | | Cannabis | | lifetime use = 13 (18.6%) | | NA | | NA | |
| Adebowale et al. (2013) | To determine the knowledge, attitude, and practices related to  drug abuse | | Secondary school students  N = 398  Males = 180 (45.2%)  Females = 218 (54.8%)  *Mean age* = 13.8±1.9 years | | Kosofe, Lagos State/Western | | Author’s developed structured questionnaire /Yes | | Indian hemp | | 14 (3.5%) | | NA | | Both were studied for substance use, so findings are not specific to Indian hemp. | |
| Afolabi et al. (2012) | To identify drugs, prevalence and factors that influence in-school adolescents to use drugs | | Senior secondary school students  N = 782  Males = 379 (48.5%)  Females = 403 (51.5%) | | Ile-Ife, Osun State/ Western | | modified United Nations school survey on drug abuse questionnaire/ Yes | | Marijuana | | Lifetime use = 3 (1.1%) | | NA | | Motivation for substance use was studied, so findings are not specific to marijuana. | |
| Aguocha and Merenu (2023) | To examine the need for school-based substance use prevention | | Junior secondary school students  N = 300  Males = 212 (70.7%)  Females = 88 (29.3%)  *Mean age*= 15.04±1.68 years | | Orlu, Imo State/Eastern | | Authors questionnaire/Yes | | Indian hemp | | past year use = 35 (11.7%) | | NA | | NA | |
| Abdullahi Hamzat and Kehinde Kanmodi (2019) | To determine the kinds of psychoactive substances used among commercial drivers | | Commercial drivers  N = 280 (Males Only)  Mean age = 40.63±10.09 years | | Sokoto, Sokoto State/Northern | | Author’s developed structured questionnaire/Yes | | Marijuana | | 45 (16.1%) used cannabis two weeks prior to study | | NA | | Both were studied for substance use, so findings are not specific to marijuana . | |
| Abdulmalik et al. (2009) | To determine the prevalence and pattern of psychoactive  substance use | | Almajiris  N = 340 (Males Only)  5 – 16 years  Mean age = 11.2±3 years | | Maiduguri, Borno Stat/ Northern | | Adapted version of the WHO Student Drug Use Questionnaire/Yes | | Cannabis | | 63 (18.5%) | | Being older (≥10 years); from a polygamous home; parental separation or death; and loneliness | | NA | |
| Abiama et al. (2014) | To investigate the pattern of substance use | | Psychiatric patients  N = 124  Males = 77 (62.1%)  Females = 47 (37.9%)  18 – 52 years  Mean age = 32.72 years | | Uyo, Akwaibom State/ Southern | | Adapted version of the WHO student Drug Use Questionnaire/Yes | | Cannabis | | current use = 35 (28.3%) | | NA | | Motivation for substance use was studied, so findings are not specific to cannabis. | |
| Abikoye et al. (2014) | To examine co-occurrence of  study difficulty and psychoactive substance use | | University students  N = 600 (3^rd^ and 4^th^ year)  Males = 295 (49.2%)  Females = 305 (50.8%)  18 – 41 years.  Males mean age = 23.9±3.39 years  Females mean age = 22.4±2.93 years | | Gwagwalada, Abuja/Northern | | WHO Student Drug Use Questionnaire/Yes | | Cannabis | | lifetime use = 87 (14.50%)  previous 12 months 73 (12.20%)  previous 30 days use = 54 (9.00%) | | Study difficulty | | NA | |
| Aguocha and Nwefoh (2021) | To assess the prevalence and socio-demographic correlates of psychoactive substance use | | University undergraduates  N= 763  Male = 323 (42.3%)  Female = 440 (57.7%)  18 – 30 years | | Owerri, Imo State/ Eastern | | modified WHO Model core student questionnaire/Yes. | | Cannabis | | lifetime use = 58 (7.6%)  12 month use = 37 (4.8%)  30 days use = 37 (4.8%)  7 days use = 27 (3.5%) | | Studied for substance use, so was not specific to cannabis | | NA | |
| Aigbogun et al. (2024) | To examine the association between certain socio-demographic characteristics and  substance use | | Internally displaced person  N = 520  Males = 124 (23.8%)  Females = 396 (76.2%) | | Maiduguri, Borno state/ Northern | | Drug use disorder identification test (DUDIT) | | Marijuana | | 31 (5.96%) | | Studied for substance use, so findings are not specific to marijuana | | Motivation for substance use was studied, so findings are not specific to marijuana | |
| Alti-Muazu and Aliyu (2008) | To determine the prevalence, health, and social consequences of psychoactive substance use | | Commercial motocyclists  N= 200 (Males only)  *Mean age* = 25.4±3.9 years | | Zaria, Kaduna State/ Northern | | Author’s developed structured questionnaire /Yes | | Marijuana (Indian hemp) | | Past year use = 52 (25.8%) | | NA | | Motivation for substance use was studied, so findings are not specific to marijuana | |
| Aluh et al. (2024) | To examine predictors and types of substances used, prevalence of substance dependence, and reasons for substance use. | | Internally displaced person  N = 520  Males = 124 (23.8%)  Females = 396 (76.2%) | | Maiduguri, Borno state/ Northern | | Drug use disorder identification test (DUDIT) | | Marijuana | | 31 (5.96%) | | Studied for substance use, so findings are not specific to marijuana | | Motivation for substance use was studied, so findings are not specific to marijuana | |
| Aniebue and Okonkwo (2008) | To ascertain the prevalence and nature of psychoactive drug use | | Taxi drivers N = 192 (Males only)  18 – 70 years  *Mean age* = 37.9±9.1 years | | Enugu State/ Southern | | Author’s developed structured questionnaire /yes | | Cannabis (Indian Hemp, Marijuana) | | 11 (3.2%) | | Studied for substance use, so findings are not specific to cannabis | | Both were studied for substance use, so findings are not specific to cannabis. | |
| Aniemena et al. (2021) | To determine the prevalence of substance use | | In-school and out-of-school adolescents N = 500  In school (n = 250)  Males = 138 (55.2%)  Females = 112 (44.8%)  *Mean age* = 16.14±1.9 years  Out-of-school (n = 250)  Males = 199 (79.6%)  Females = 51 (20.4%)  *Mean age* = 16.68±1.71years  (10 – 19 years) | | Onitsha, Anambra State/ Eastern | | adapted from WHO instrument on  drug use among non-student youth | | Cannabis | | In-school  ever use = 19 (7.6%)  past 12 months = 10 (58.8%)  nearly every week = 7 (36.8%)  nearly every day = 4 (21.1%)  Out-of-school  ever use = 89 (35.6%)  past 12 months = 82 (91.1%)  nearly ever week = 30 (33.3%)  nearly every day = 48 (53.3%) | | NA | | NA | |
| Apetuje Emmanuel Temidayo (2018) | To explore the prevalence of drug use and abuse | | Secondary school students and university undergraduates  N = 180  Males = 99 (55%)  Females = 81 (45%)  12 – 44years | | Oye Ekiti, Ekiti State/ Western | | Author’s developed structured questionnaire/Yes | | Marijuana  Hashish | | Ever use = 26 (14.6%)  Ever use = 1 (0.6%) | | Studied for drug use, so findings are not specific to marijuana | | Motivation for drug use was studied, so findings are not specific to marijuana | |
| Atilola et al. (2013) | To determine the prevalence, pattern and sociodemographic correlates -of alcohol/substance use | | Senior secondary school students N = 538  Males = 244 (44.4%)  Females = 294 (55.6%)  Mean age 15.1±1.4 years | | Ibadan, Oyo State/ Western | | CRAFFT/Yes | | Marijuana | | 12 months use = 5 (1.0%) | | Studied for substance use, so findings are not specific to marijuana | | NA | |
| Babalola et al. (2014) | To determine the prevalence, pattern and factors associated with psychoactive substance use | | University students  N = 246 ( Medical students)  Males = 130 (52.8%)  Females = 116 (47.7%)  Mean age = 26.8 years | | Ago-Iwoye, Ogun State/ Western | | WHO Student Drug Use Questionnaire/Yes | | Cannabis | | lifetime use = 13 (5.3%)  previous year use = 13 (5.3%)  current use = 11 (4.5%) | | A significant proportion of males than females reported lifetime use | | NA | |
| Dimas et al. (2021) | To examine the prevalence of substance abuse and its determinant factors | | Substances users N = 198  Males = 140 (70.7%)  Females = 58 (29.3%) | | Jigwada, Nassarawa State/ Northern | | Substance Use/Abuse Prevalence Inventory (SUAPI)/Yes | | Marijuana | | 108 (54.5%) | | Studied for substance use, so findings are not specific to marijuana | | NA | |
| Durowade et al. (2021) | To assess the prevalence, predictors, patterns, and types of substance use | | University students  N = 416  Males = 188 (45.2%)  Females = 228 (54.8%) | | Ado Ekiti, Ekiti State/ Western | | Author’s developed structured questionnaire/Yes | | Marijuana | | current use = 12 (4.0%) | | Studied for substance use, so findings are not specific to marijuana | | NA | |
| Ejikem et al. (2023) | To evaluate the patterns of psychoactive substance use and determinants. | | Adolescents  N = 1036  10 – 19 Years | | Abia State/Eastern | | Author’s developed structured questionnaire/Yes | | Cannabis | | First substance used  Rural = 70 (12.8%)  Urban = 68 (13.8%)  Regularly  Rural = 220 (64.3%)  Urban = 206 (67.1%)  Occasionally  Rural = 67 (19.6%)  Urban = 61 (19.9%) | | Studied for substance use, so findings are not specific to cannabis | | NA | |
| Ekop et al. (2019) | To determine the prevalence and pattern of substance use | | Adolscents  N = 1196  Males = 555 (46.4%)  Females = 641 (53.6%) | | Gwagwalada, Abuja/Northern | | Author’s developed structured questionnaire/Yes | | Cannabis | | Lifetime use = 17 )1.4%)  Past 12 months use = 14 (1.2%)  Use within 30 days = 9 (0.8%) | | Studied for substance use, so findings are not specific to cannabis | | NA | |
| Ekwueme & Chukwuneke (2010) | To determine pattern of psychoactive substance use, mental and behavioral effect | | University students  N = 422 | | Enugu, Enugu State/Eastern | | Author’s developed structured questionnaire/Yes | | Marijuana | | 6 (1.4%) | | Studied for substance use, so findings are not specific to marijuana | | NA | |
| Ipingbemi & Akerele (2021) | To determine prevalence of psychoactive substance use, awareness of health risks and associated financial expenditure | | University students  N = 521  Public: Males = 183 (57.9%)  Females 132 (41.8%)  Private: Males = 69 (33.7%)  Females = 136 (66.3%)  Mean age = 20.5±2.4 years | | Ibadan and Iwo, Oyo State/Western | | WHO Alcohol, smoking and substance involvement screening test v3.0/Yes | | Cannabis | | Public: Use within 30 days  = 8 (2.5%)  Lifetime = 7 (2.2%)  Private:  Use within 30 days  = 5 (2.4%)  Lifetime = 4 (2.0%) | | Studied for substance use, so findings are not specific to cannabis | | Motivation for drug use was studied, so findings are not specific to cannabis | |
| Eniojukan (2015) | To determine the prevalence and patterns of cigarette smoking | | Secondary school students  N = 1149 (Junior school)  Males = 662 (57.7%)  Females = 487 (42.5%) | | Benue state/Northern | | Author’s developed structured questionnaire/Yes | | Cannabis | | Current use = 6 (8.3%) | | Studied for substance use, so findings are not specific to cannabis | | Both were studied for substance use, so findings are not specific to marijuana | |
| Erinoso et al. (2021) | To assess the prevalence and factors associated with electronic cigarette use and anxiety | | Secondary school and university students  N = 949  Males = 421 (44.4%)  Females = 528 (55.6%)  Age = 15 – 35 years  Mean age = 23.26±3.97 years | | Lagos state/Western | | Author’s developed structured questionnaire/Yes | | Cannabis | | Ever use = 6 (8.1%) | | Studied for substance use, so findings are not specific to cannabis | | NA | |
| Ezema et al. (2022) | To explore psychoactive substance use and its effect on treatment outcome | | People living with HIV (PLWHIV)  N = 700  Male = 259 (37%)  Females = 441 (63%)  Mean age = 39.3±10.1 years | | Makurdi, Benue State/Northern | | WHO ASSIST/Yes | | Cannabis | | Current use  Once/twice = 4 (0.6%)  Last 3 months = 6 (0.9%) | | Studied for substance use, so findings are not specific to cannabis | | NA | |
| Fatoye & Morakinyo (2002) | To determine the prevalence and pattern of drug use | | Secondary school students  N = 567  Males = 266 (47.3%)  Females = 276 (52.7%)  *Mean age* = 17±1.69 years | | Ilesa, Osun State/Western | | WHO Student Drug Use Questionnaire/Yes | | Cannabis | | Lifetime use = 3 (0.5%)  Past 12 months use = 2 (0.4%)  Current use = 1 (0.2%) | | NA | | NA | |
| Fela-Thomas et al. (2020) | To ascertain prevalence of psychoactive substance use, misuse of prescription medications and level of risk | | Primary health care patients, N = 649  Males = 371 (57.2%)  Females = 277 (42.7%)  14 – 59 years  Mean age = 37.5±11.55 years | | Benin city, Edo State/Southern | | WHO ASSIST/Yes | | Marijuana | | Lifetime use = 33 (5.1%)  Current use = 13 (2.0%) | | Males used marijuana daily or weekly in the last three months. | | NA | |
| Fela-Thomas et al. (2019) | To assess prevalence, correlates of psychoactive substance use, misuse of prescription medications and associated harm | | Elderly patients  N = 173  Males = 90 (52%)  Females = 83 (48%)  60 – 89 years | | Benin city, Edo State/Southern | | WHO ASSIST/Yes | | Marijuana | | Lifetime use = 2 (1.2%)  Current use = 1 (0.6%) | | Studied for substance use, so findings are not specific to cannabis | | NA | |
| Gureje et al. (2007) | To examine psychoactive substance use and dependence | | Adults  N = 6752  Males = 3307 (69.4%)  Females = 3445 (46.5%)  Mean age = 18 – 65+ years | | States in all 4 regions/ | | WHO CIDI/Yes | | Cannabis | | Lifetime use = 2.7%  Past year use = 0.4% | | Being a male | | NA | |
| Hamzat et al. (2019) | To determine the prevalence of the use of stimulants, narcotics, and hallucinogens | | Long-distance commercial drivers  N = 280  Males only  Mean age = 40.63±10.09 years | | Sokoto city, Sokoto State/Northern | | Author’s developed structured questionnaire/Yes | | Marijuana | | Past 2 weeks = 45 (16.1%) | | NA | | NA | |
| Hassan & Afolaranmi (2014) | To determine pattern of substance use | | People living with HIV  N = 70  Males = 37 (52.9%)  Females = 33 (47.1%)  18 – 49 years  Mean age = 30±2.03 years | | Jos north LGA, Plateau State/Northern | | Author’s developed structured questionnaire/Yes | | Marijuana | | Ever or current use = 4 (5.7%) | | Studied for substance use, so findings are not specific to cannabis | | NA | |
| Idowu et al. (2018) | To assess the prevalence and factors associated with substance abuse | | Secondary school students  N = 249  Males = 127 (51%)  Females = 122 (49%)  Mean age = 16.3±2 years | | Ogbomoso LGA, Oyo State/Western | | Author’s developed structured questionnaire/Yes | | Cannabis | | Ever use = 3 (1.0%) | | Studied for substance use, so findings are not specific to cannabis | | Motivation for drug use was studied, so findings are not specific to cannabis | |
| Idris & Sambo (2009) | To determine pattern of use and the associated factors | | Secondary school students  N = 280  Males = 195 (69.64%)  Females = 85 (30.36%)  13 – 18+ years | | Zaria LGA, Kaduna/Northern | | Author’s developed structured questionnaire/Yes | | Cannabis | | 20 (7.1%) | | Studied for substance use, so findings are not specific to cannabis | | NA | |
| Igwe & Ojinnaka (2010) | To determine the prevalence of psychosocial dysfunction and depressive symptoms | | Adolescents who use drugs  N = 860  Males = 499 (57.4%)  Females = 360 (42.6%)  10 – 19 years  16.9±1.7 years | | Enugu, Enugu State/Eastern | | WHO Student Drug Use Questionnaire/Yes | | Cannabis | | Current use = 35 (4.10%) | | Studied for substance use, so findings are not specific to cannabis | | NA | |
| Oderinde et al. (2020) | To evaluate co morbidity and associated clinico-demographic characteristics  Associated with diagnoses of substance use disorder | | Psychaitric patients  N = 88  Males = 85 (96.6%)  Females = 3 (3.4%)  10 – 50+ years | | Damaturu, Yobe State/ Northern | | Author’s developed structured questionnaire/Yes | | Indian Hemp | | Daily Use = 36 (40.9%) | | Studied for substance use, so findings are not specific to cannabis | | - To feel high - Compulsive urge - To improve mood - To prevent withdrawal synydromes - To prolong time of sexual intercourse | |
| Koyejo & Gbiri (2015) | To determine prevalence, pattern and psychoactive substance used | | People living with HIV  N = 386  Males = 129 (33.51%)  Females = 256 (66.49%)  15 – 60+ years  Mean age = 37.62±9.16 years | | Ikeja, Lagos State/Western | | Author’s developed structured questionnaire/Yes | | Cannabis | | Life time use = 14 (3.64%)  Current use = 3 (0.78%) | | NA | | NA | |
| Lasebikan & Adebayo (2013) | To examine the prevalence of drug use and abuse, identify socio-demographic characteristics and risk factors correlated with injury. | | Trauma patients  N = 1121  Males = 594 (88.1%)  Females = 526 (11.9%) | | Ibadan, Oyo state/Western | | WHO CIDI/Yes. | | Cannabis | | Injured  Past 12 months use = 499 (44.5%)  Past 12 months abuse = 157 (14%)  Control  Past 12 months use = 110 (36.3%)  Past 12 months abuse = 32 (10.6%) | | Severity of injury | | NA | |
| Lasebikan & Ijomanta (2019) | To investigate the prevalence of drug use | | Military officers  N = 223  Mean age = 38  Age = 24 – 58 years | | Unspecied | | WHO CIDI/Yes | | Cannabis | | 12 months use = 5 (33.3%) | | Studied for substance use, so findings are not specific to cannabis | | NA | |
| Lawoyin et al. (2005) | To determine the prevalence of drug use and associated socio-demographic factors | | Senior secondary school students  N = 394  Males = 217  Females = 177  Age = 14 – 24 years | | Igboora, Oyo State/Western | |  | | Hashish | | Ever use = 2 (0.7%) | | Studied for substance use, so findings are not specific to cannabis | | NA | |
| Manyike et al. (2016) | | To determine the prevalence and pattern of psychoactive substance use | | Secondary school students  N = 896  Mean age = 15.9±1.04 years  Age = 15 – 19 years | | Enugu metropolis, Enugu State/Eastern | | WHO student drug use questionnaire/Yes | | Cannabis | | Past year use = 7 (0.8%)  Lifetime use = 7 (0.8%)  Current use = 4 (0.4%) | | Studied for substance use, so findings are not specific to cannabis | | NA |
| Morakinyo & Odejide (2003) | | To determine the pattern of psychoactive substance use and associated socio-demographic and street factors. | | Street children  N = 180  Male = 174 (96.7%)  Females = 6 (3.3.%)  Mean age = 14.6±2.6 years  Age = 8 – 18 years | | Ibadan North, Oyo State/ Western | | Pimrat-Awele Drug Questionnaire | | Cannabis | | Lifetime use = 10%  Current use = 14 (7.8%) | | Studied for substance use, so findings are not specific to cannabis | | Motivation for the use of substances and the Source of the substances were studied, so findings are not specific to cannabis. |
| Musa et al. (2021) | | To assess the level of knowledge and prevalence of drugs and substance abuse | | University students  N = 151  Male = 97 (64.2%)  Mean age = 27.3±6.2 years | | Kano, Kano State/Northern | | Author’s questionnaire/Yes | | Marijuana | | 4 (14.3%) | | Studied for substance use, so findings are not specific to cannabis | | Motivation for drug ause was studied, so findings are not specific to marijuana |
| Njoku & Obogo (2017) | | To investigate the relationship between depression and drug abuse. | | Secondary school students  N = 200 | | Calabar, Cross River State/Southern | | Drug Abuse Screening Test/Yes | | Marijuana | | 4 (10%) | | Studied for substance use, so findings are not specific to marijuana | | NA |
| Nyango et al. (2012) | | To determine what substances are used and the prevalence of substance use | | Pregnant women  N = 557  Age = 15 – 48 years  Mean age = 29.2±5.3 years | | Jos, Plateau State/Northern | | Author’s questionnaire/Yes | | Marijuana | | Past one month = 9 (1.6%) | | NA | | Motivation for drug ause was studied, so findings are not specific to marijuana |
| Obadeji et al. (2020) | | To identify the pattern of substance use among high school students and its relationship with psychosocial factors | | Secondary school students  N = 682 (Senior school)  Males 391 (57.3%)  Females = 291 (42.7%)  Age = 13 – 19 years  Mean age = 15.75±1.35 years | | Ado Ekiti, Ekiti State/Western | | Author’s questionnaire/Yes | | Cannabis | | Lifetime use = 15 (2.2%)  Current use = 15 (2.2%) | | Being a male | | NA |
| Oderinde et al. (2020) | | To evaluate the co morbidity and associated clinico-demographic characteristics  of patients diagnosed with substance use disorder | | Psychiatric patients  N = 88  Males = 85 (96.6%)  Females = 3 (3.4%) | | Damataru, Yobe State/Northern | | Author’s questionnaire/Yes | | Indian hemp | | Daily Use = 40.9% | | Peer influence and curiosity | | - Prolonged time of sexual intercourse - To improve mood - To feel high   Compulsive urge and to relieve tiredness |
| Odukoya et al. (2018) | | To assess the relationship between parental monitoring practices and drug use | | Secondary school students  N = 437 (Senior School)  Males = 204 (46.7%)  Females = 233 (53.3%)  Age = 10 – 19 years  Mean age = 15.30±1.60 years | | Mushin, Lagos State/Western | | Youth risk behavior surveillance system questionnaire/Yes | | Marijuana | | Lifetime Use = 86 (19.7%) | | Negotiated unsupervised time | | NA |
| Johnson et al. (2017) | | To determine the prevalence and factors predisposing to psychoactive substance use | | University undergraduates  N = 324  Males = 170 (52.5%)  Females = 154 (47.5%)  Age = 18 – 25 years  Mean age = 21.57±1.96 years | | Uyo, AkwaIbom state/Southern | | Author’s questionnaire/Yes | | Marijuana | | Lifetime Use = 28 (31.5%) | | Studied for substance use, so findings are not specific to cannabis | | Motivation for substance use was studied, so findings are not specific to marijuana |
| Ojieabu et al. (2015) | | To assess substance abuse | | Young adults  N = 300  Males = 149 (49.7%)  Females = 151 (50.3%)  Age = 15 – 50 years | | Sagamu, Ogun State/Western | | Author’s questionnaire/Yes | | Indian hemp | | Lifetime Use = 51 (17%) | | NA | | Motivation for substance abuse was studied, so findings are not specific to Indian hemp |
| Ojule & Te-Erebe (2022) | | To examine prevalence and predictors of substance use disorder | | Secondary school students  N = 384  Males = 195 (50.8%)  Females = 189 (49.2%)  Age = 10 – 25 years | | Khana, Rivers State/Southern | | Author’s questionnaire/Yes | | Indian hemp | | Urban = 15 (16.7%)  Rural = 16 (19.8%) | | Studied for substance use, so findings are not specific to cannabis | | Source of substance was studied, so findings are not specific to Indian hemp |
| Okpataku et al. (2015) | | To determine medication adherence behavior among psychiatric out-patients with psychoactive substance use comorbidity | | Psychiatric patients  N = 208  Males = 110 (52.9%)  Females = 98 (47.1%)  Mean age = 36.72±12.69 years | | Zaria, Kaduna State/Northern | | Drug Use questionnaire/Yes | | Cannabis | | Lifetime Use = 27 (13%) | | NA | | NA |
| Olanrewaju et al. (2022) | | To assess the prevalence and awareness of drug and substance abuse | | University students  N = 400  Males = 128 (32%)  Females = 272 (68%)  Age = ≥15 years | | Ekiti, Osun, Oyo, Ondo and Lagos states/Western | | Author’s questionnaire/Yes | | Cannabis | | Lifetime Use = 102 (25.5%) | | NA | | Motivation for substance abuse was studied, so findings are not specific to cannabis |
| Ogunwale et al. (2012) | | To examine psychoactive substance use among young offenders | | Borstal inmates = 54  Secondary school students = 104 | | Abeokuta, Ogun State/Western | | WHO Students Drug Use Questionnaire/Yes | | Cannabis | | Inmates  Lifetime use = 26 (28.1%)  Past year use = 13 (24.1%)  School students  Current use 1(1.9%) | | NA | | NA |
| Omotoso et al. (2020) | | To determine the prevalence and pattern of substance use | | Secondary school students  N = 2001  Males = 1083 (54.1%)  Females = 918 (45.9%)  Mean age = 15.05±2.239 | | Ilorin, Kwara State/Northern | | WHO Students Drug Use Survey Questionnaire/Yes | | Cannabis | | Lifetime use = 239 (11.9%)  Current use = 12 (0.6%) | | Fathers use of cannabis | | NA |
| Osalusi et al. (2022) | | To investigate the prevalence of psycho active substance use and associated risk factors | | University undergraduates  N = 347  Males = 270 (69.77%)  Females = 117 (30.23%)  Age = 14 – 30 years  Mean age = 20.51±2.91 years | | Akoka, Lagos State/Western | | WHO Students Drug Use Survey Questionnaire/Yes | | Cannabis/Marijuana | | Ever use = 51 (13.18%)  Past 12 months use = 34 (8.79%)  Past one month use = 27 (6.98%) | | Studied for substance use, so findings are not specific to cannabis | | NA |
| Oshodi et al. (2010) | | To establish the prevalence and associated factors of substance use | | Secondary school students  N = 402  Males = 175 (43.5%)  Females = 227 (56.5%)  Age = 11 – 20 years  Mean age = 15.9 years | | Surulere, Lagos State/Western | | WHO Students Drug Use Survey Questionnaire/Yes | | Cannabis | | Lifetime use = 16 (4.4%)  Past year use = 4 (1.1%)  Current use = 12 (3.3%) | | Studied for substance use, so findings are not specific to cannabis | | Motivation for substance abuse was studied, so findings are not specific to cannabis |
| Oyapero et al. (2022) | | To explore the relationship between stress perception and self-reported lifestyles | | Commercial drivers  N = 200  Males = 193 (96.5%)  Females = 7 (3.5%)  Mean age = 42.70±10.5 years | | Ojota and Berger, Lagos State/Western | | Health related quality of life/Yes | | Cannabis | | Past year use = 5%  Current use = 44%  Daily use = 20% | | NA | | NA |
| Oye-Adeniran et al. (2014) | | To examine substance use and associated sexual risk behaviors | | University students  N = 2408 Females  University of ibadan = 1864  Bayero University = 554  Age = 17 – 49 years  Mean age = 21.6±2.9 years | | Ibadan, Oyo State and Kano, Kano State/Western and Northern | | Authors questionnaires/Yes | | Cannabis | | 21 (1%) | | NA | | NA |
| Shehu & Idris (2008) | | To determine the prevalence and the factors associated with smoking and its effects on academic performance | | Secondary school students  N = 350  Males = 262 (74.9%)  Females = 88 (25.1%) | | Zaria Local government area, Kaduna State/Northern | | Author’s questionnaire/No | | Marijuana | | Ever use = 33 (9.4%) | | Age | | - Increase confidence - Relaxation - Happiness |
| Shuaibu et al. (2024) | | To determine the prevalence, risk, and protective factors of substance use | | Adolescents  N = 298  Males = 112 (37.6%)  Females = 186 (62.1%)  Age = 10 – 19 years  Mean age = 13.5±2.3 years | | Abuja/Northern | | Youth Risk Behaviour Survey/Yes | | Indian hemp | | Lifetime use = 7 (2.3%)  Current use = 7 (2.3%) | | Studied for substance use, so findings are not specific to Indian hemp | | NA |
| Soremekun et al. (2020) | | To measure the prevalence of drug use | | Secondary school students  N = 1048  Males = 533 (50.86%)  Females = 515 (49.14%) | | Ikotun/Igando and Ikoyi, Lagos State/ Western | | Author’s questionnaire/Yes | | Cannabis | | Lifetime use = 2.4%  Current use = 1% | | Senior secondary class and living in lower economic areas | | NA |
| Soremekun et al. (2021) | | To determine the  prevalence of drug abuse | | University students  N = 850  Federal = 464  State = 303  Private = 60 | | Ekiti, Ekiti State/Western | | ASSIST/Yes | | Marijuana | | 15.7% | | Being in final year (400 level) and a state university | | NA |
| Sulyman et al. (2020) | | To examine the use of psychoactive substances | | University undergraduates  N = 983  Males = 560 (57%)  Females = 423 (43%)  Age = 17 – 38 years  Mean age = 23.3±3.4 years | | Bauchi, Bauchi State/Northern | | WHO Student drug use survey questionnaire/Yes | | Cannabis | | Current use = 4.5% | | Studied for psychoactive substance use, so findings are not specific to cannabis | | NA |
| Ugwuoke & Ifeanyichukwu (2016) | | To investigate drug use pattern | | Prison inmates  N = 3134  Males = 3112 (99.3%)  Females = 22 (0.7%)  Mean age = 19 – 60 years (34.4) | | Jos, Plateau State/Northern | | Author’s questionnaire/Yes | | Cannabinoids (Hashisha and Marijuana) | | 2108 (67.3%) | | NA | | NA |
| Unaogu et al. (2017) | | To identify the common drugs of abuse, socio-demographic features and clinical characteristics of individuals who abuse substance | | Psychiatric patients  N = 86  Males = 83 (96.5%)  Females = 3 (3.5%)  Mean age = 30.88±8.49 years | | Enugu, Enugu State/Eastern | | Authors questionnaire/Yes | | Cannabis | | 70 (81.4%) | | NA | | NA |
| Abubakar et al. (2021) | | To examine knowledge of health effects and determinants of psychoactive substance use | | Secondary school students  N = 430  Males = 284 (68.4%)  Females = 146 (31.6%)  Mean age = 16.3±3.1 years | | Sokoto metropolis, Sokoto State/Northern | | Authors questionnaire/Yes | | Cannabis | | Current use = 15 (21.4%) | | Studied for psychoactive substance use, so findings are not specific to cannabis. | | NA |
| Wada et al. (2021) | | To assess the prevalence and impacts of psychoactive substance abuse | | University undergraduates  N = 308  Males = 207 (67.2%)  Females = 101 (32.8%) | | Katsina, Katsina State/Northern | | Authors questionnaire/Yes | | Cannabis | | 38 (13.3%) | | NA | | Motivation for substance abuse was studied, so findings are not specific to cannabis. |
